# CRAC: an integrated approach to the analysis of RNA-seq reads

**DOI:** 10.1186/gb-2013-14-3-r30

**Published:** 2013-03-28

**Authors:** Nicolas Philippe, Mikaël Salson, Thérèse Commes, Eric Rivals

**Affiliations:** 1 Laboratoire d'Informatique, de Robotique et de Microélectronique de Montpellier (LIRMM), UMR 5506, CNRS and Université de Montpellier 2, 161 rue Ada, 34095 Montpellier Cedex 5, France; 2 Institut de Recherche en Biothérapie (IRB), U1040 INSERM, CHRU Montpellier Hôpital Saint-Eloi 80, av. Augustin Fliche, 34295 Montpellier Cedex 5, France; 3 Laboratoire d'Informatique Fondamentale de Lille (LIFL), (UMR CNRS 8022, Université Lille 1) and Inria Lille-Nord Europe, Cité scientifique-Bâtiment M3, 59655 Villeneuve d'Ascq Cedex, France; 4 LITIS EA 4108, Université de Rouen, 1 rue Thomas Becket, 76821 Mont-Saint-Aignan Cedex, France; 5 Institut de Biologie Computationnelle, 95 Rue de la Galéra, 34095 Montpellier Cedex 5, France

## Abstract

A large number of RNA-sequencing studies set out to predict mutations, splice junctions or fusion RNAs. We propose a method, CRAC, that integrates genomic locations and local coverage to enable such predictions to be made directly from RNA-seq read analysis. A *k*-mer profiling approach detects candidate mutations, indels and splice or chimeric junctions in each single read. CRAC increases precision compared with existing tools, reaching 99:5% for splice junctions, without losing sensitivity. Importantly, CRAC predictions improve with read length. In cancer libraries, CRAC recovered 74% of validated fusion RNAs and predicted novel recurrent chimeric junctions. CRAC is available at http://crac.gforge.inria.fr.

## Rationale

Understanding the molecular processes responsible for normal development or tumorigenesis necessitates both identifying functionally important mutations and exploring the transcriptomic diversity of various tissues. RNA sequencing (RNA-seq) provides genome-scale access to the RNA complement of a cell with unprecedented depth, and has therefore proven useful in unraveling the complexity of transcriptomes [1,2]. The analyses of RNA-seq reads aim at detecting a variety of targets: from transcribed exons and classical splice junctions with canonical splice sites, to alternatively spliced RNAs, RNAs with non-standard splice sites, read-through and even non-colinear chimeric transcripts [3]. Moreover, RNA-seq also gives access to those somatic mutations and genetic polymorphisms that are transcribed. Chimeric RNAs result from the transcription of genes fused together by chromosomal rearrangements [4], especially in cancer [5], and they can also be induced by trans-splicing between mature messenger RNAs (mRNAs) [6]. RNA-seq can also capture these complex, non-colinear transcripts, whose molecular importance is still poorly assessed and which may provide new diagnostic or therapeutic targets [7,8].

As next generation sequencing (NGS) improves and becomes cheaper, bioinformatic analyses become more critical and time consuming. They still follow the same paradigm as in the first days of NGS technologies: a multiple step workflow - mapping, coverage computation, and inference - where each step is heuristic, concerned with only a part of the necessary information, and is optimized independently from the others. Consequently analyses suffer from the drawbacks inherent to this paradigm: (a) pervasive erroneous information, (b) lack of integration, and (c) information loss, which induces re-computation at subsequent steps and prevents cross-verification. An example of the lack of integration is that the mapping step cannot use coverage information, which prevents it from distinguishing biological mutations from sequencing errors early in the analysis.

Here, we design a novel and integrated strategy to analyze reads when a reference genome is available. Our approach extracts information solely from the genome and read sequences, and is independent of any annotation; we implemented it in a program named CRAC. The rationale behind it is that an integrated analysis avoids re-computation, minimizes false inferences, and provides precise information on the biological events carried by a read. A peculiarity of CRAC is that it can deliver computational predictions for point mutations, indels, sequence errors, normal and chimeric splice junctions, in a single run. CRAC is compared with state-of-the-art tools for mapping (BWA, SOAP2, Bowtie, and GASSST) [9-13], and both normal (GSNAP, TopHat, and MapSplice) [3,14,15] and chimeric (TopHat-fusion) [16] splice junction predictions. The results show the relevance of the approach in terms of efficiency, sensitivity, and precision (which is also termed specificity in the literature). We also provide true assessments of the sensitivity of each method by analyzing complex simulated data.

Availability: CRAC is distributed under the GPL-compliant CeCILL-V2 license and is available as source code archive or a ready-to-install Linux package from the CRAC project website [17] or the ATGC bioinformatics platform [18]. It includes two programs: crac-index to generate the index of the genome, and crac for analyzing the reads.

## Algorithm

### Overview

CRAC is a method for analyzing reads when a reference genome is available, although some procedures (for example, the support computation) can be used in other contexts as well. CRAC analysis is solely based on the read collection and on the reference genome, and is thus completely independent of annotations. CRAC disregards the sequence quality information of reads. Here, analyzing reads means detecting diverse biological events (mutations, splice junctions, and chimeric RNAs) and sequencing errors from a RNA-seq read collection.

CRAC analysis is based on two basic properties: P1 and P2.

P1: For a given genome size, a sequence of a specific length will match on average to a unique genomic position with high probability. This length, denoted *k*, can be computed and optimized [19]. Thus, in a read any *k*-mer (a *k*-long substring) can be used as a witness of the possible read matching locations in the genome. A *k*-mer may still have a random match to the reference genome. However, in average over all *k*-mers, the probability of getting a false location (FL) is approximately 10^−4 ^with *k *= 22 for the human genome size [19].

P2: As reads are sequences randomly sampled from biological molecules, several reads usually overlap a range of positions from the same molecule. Hence, a sequencing error that occurs in a read should not affect the other reads covering the same range of positions. In contrast, a biological variation affecting the molecule should be visible in many reads overlapping that position.

CRAC processes each read in turn. It considers the *k*-mers starting at any position in the read (that is, *m *- *k *+ 1 possible *k*-mers). It computes two distinct *k*-mer profiles: the location profile and the support profile.

• The **location profile **records for each *k*-mer its exact matching locations on the genome and their number.

• The **support profile **registers for each *k*-mer its support, which we define as the number of reads sharing this *k*-mer (that is, the *k*-mer sequence matches exactly a *k*-mer of another read). The support value has a minimum value of one since the *k*-mer exists in the current read.

CRAC's strategy is to analyze these two profiles jointly to detect multiple events and predict sequencing errors in a single analysis, as well as potential genetic variations, splice junctions, or chimeras (Additional file 1). The genomic locations of a *k*-mer are computed using a compressed index of the reference genome, such as a compressed Burrows-Wheeler transform [20], while the support of a *k*-mer is obtained on-the-fly by interrogating a specialized read index, called a Gk arrays [21]. CRAC ignores the pairing information of paired end reads. Each read in a pair is processed independently of the other.

Clearly, the support is a proxy of the coverage and allows property P2 to be exploited for distinguishing sequencing errors from variations, and gaining confidence in predictions. As illustrated below, the location profile delivers a wealth of information about the mapping, but the originality of CRAC is its ability to detect the concordance of variations in the two profiles.

### Description of the algorithm

In a collection, some reads will exactly match the reference genome, while others will be affected by one or more differences (with a probability that decreases with the number of differences). Here, we describe how a read is processed and concentrate on reads that differ from the reference. For clarity, we make simplifying assumptions: (a) *k*-mers have no false genomic locations, (b) the read is affected by a single difference (substitution, indel, or splice junction), and (c) this difference is located >*k *nucleotides away from the read's extremities (otherwise, we say it is a border case). These assumptions are discussed later.

**Figure 1:**
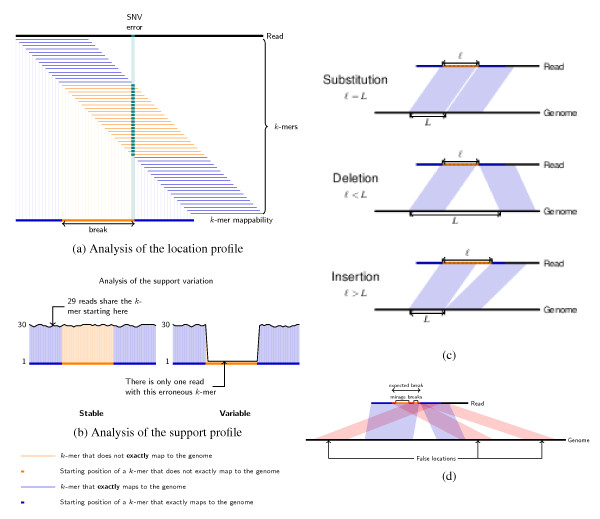
**The CRAC algorithm**. **(a) **Illustration of a break in the location profile. We consider each *k*-mer of the read and locate it exactly on the genome. In all figures, located *k*-mers are shown in blue, and unmapped *k*-mers in light orange. If the read differs from the genome by, for example an SNV or an error, then the *k*-mers containing this position are not located exactly on the genome. The interval of positions of unmapped *k*-mers is called a break. The end position of the break indicates the error or SNV position. **(b) **The support profile. The support value of a *k*-mer is the number of reads from the collection in which this *k*-mer appears at least once. The two plots show the support profile as a black curve on top of the location profile (in blue and orange). The support remains high (left plot) over the break if many reads covering this region are affected by a biological difference (for example, a mutation); it drops in the region of the break when the analyzed read is affected by a sequencing error; in this case, we say the support is dropping. **(c) **Rules for differentiating a substitution, a deletion, or an insertion depending on the break. Given the location profile, one can differentiate a substitution, a deletion, or an insertion by computing the difference between the gap in the genome and the gap in the read between *k*-mers starting before and after the break. **(d) **False locations and mirage breaks. When false locations occur inside or at the edges of a break they cause mirage breaks. False locations are represented in red. The break verification and break merging procedures correct for the effects of false locations to determine the correct break boundaries (and for example the correct splice junction boundaries) to avoid detecting a false chimera (Rule 2a) instead of a deletion. SNV: single nucleotide variant

Consider first a substitution, which may be erroneous (a sequencing error) or of biological origin (an SNP, single nucleotide variant (SNV), or editing). Say the substitution is at position *h *in the read. All *k*-mers overlapping position *h *incorporate this difference and will not match the genome. Thus, the location profile will have zero location for *k*-mers starting in the range [*h *- *k *+ 1, *h*]. In contrast, *k*-mers starting left (respectively right) of that range will have one location in the genome region where the RNA comes from. Moreover, locations of the *k*-mers starting in *h *− *k *and *h *+ 1 are *k *+ 1 nucleotides apart on the genome. We call the range of *k*-mers having zero location, a break (Figure 1a). This allows the location of the difference in both the read and the genome to be found, but does not distinguish erroneous from biological differences. The support profile will inform us on this matter.

If the substitution is a sequencing error, it is with high probability specific to that read. Hence, the *k*-mers overlapping the substitution occur in that read only: their support value is one (minimal). If the substitution is biological, a sizeable fraction of the reads covering this transcript position share the same *k*-mers in that region. Their support remains either similar to that of *k*-mers outside the break or at least quite high depending on the homozygosity or heterozygosity of the mutation. An erroneous difference implies a clear drop in the support profile over the break (Figure 1b). Thus, the ranges of the location break and the support drop will coincide for an error, while a biological difference will not specifically alter the support profile over the break. To detect this drop we compare the average support inside versus outside the break using a separation function (Figure 1b and Additional file 2). Using this procedure, support profiles are classified as undetermined if the support is too low all along the read, and otherwise as either dropping or non-dropping. Reads with a dropping support profile are assumed to incorporate sequencing errors, and those with a non-dropping support to accurately represent sequenced molecules.

This procedure can be generalized to differences that appear as long indels; all cases are summarized by a detection rule. We can apply a similar location/support profile analysis to predict such events.

Rule 1 (Figure 1c): Consider a read affected by a single difference (substitution, indels) compared to the genome. Let *j_b _*<*j_a _*(where *b *stands for before and *a *after) be the positions immediately flanking the observed break in the location profile (that is, the break is in the range [*j_b _*+ 1, *j_a _*− 1]). Let *l *:= *j_a _*− *j_b_*. *L *denotes the offset between the genomic locations of the *k*-mers starting in *j_b _*and *j_a_*, so that *L *:= loc(*j_a_*) - loc(*j_b_*). (1) If *l *= *L *= *k *+ 1 the difference consists of a single substitution at position *j_a _*− 1 in the read and loc(*j_a_*) − 1 in the genome. (2) If *l *= *k *and *L *= *k *+ *p *for some integer *p*, then this is a *p *nucleotide deletion with respect to the reference genome, which is located between position *j_a _*− 1 and *j_a _*in the read, and between loc(*j_a_*) − *p *and loc(*j_a_*) − 1 on the genome. (3) Symmetrically, if *l *= *k *+ *p *and *L *= *k *for some integer *p*, the difference is a *p *nucleotide insertion with respect to the reference.

We call the *k*-mer concordance the condition that loc(*j_a_*) and loc(*j_b_*) are on the same chromosome, the same strand, and that loc(*j_a_*) − loc(*j_b_*) equals *j_a _*- *j_b _*plus or minus the inferred difference (that is, 0 for a substitution and *p *for indels). This notion can be extended to all *k*-mer pairs on each side of the break (that is, not merely *j_b_*, *j_a_*).

The observed missing part in the read can be due to a polynucleotidic deletion or the removal of intronic or intragenic regions by splicing. Without annotations, only the expected length (that is, the value of *p*) can distinguish these cases. CRAC uses arbitrary, user-defined thresholds to classify such biological deletions into short deletions and splice junctions. CRAC does not use splice site consensus sequences.

Rule 2: Other reads may present profiles not considered in Rule 1. In particular, some reads will have a break but the genomic locations at its sides are either on distinct chromosomes or not colinear on the same chromosome. We term these chimeric reads (by chimeric we mean made of a non colinear arrangement of regions rather than unreal), and consider three subcases corresponding to possible known combinations [4]: (a) same chromosome, same strand but inverted order, (b) same chromosome but different strands, and (c) different chromosomes. (For chimeric RNAs, CRAC can even distinguish five subclasses; see Additional file 2 for details). CRAC can handle such cases with the profile analysis. These cases resemble that of deletions (Rule 1, case 2), except that the genomic locations are not colinear. Indeed, CRAC checks the break length *l *= *k*, as well as the coherence of adjacent *k*-mers left or right of the break. Coherence means that, for some (small) integer δ, *k*-mers in the range [*j_b _*− δ, *j_b_*] (respectively, [*j_a_*, *j_a _*+ δ]) have adjacent locations on the genome. Reads satisfying these criteria and harboring a non-dropping support profile are primarily classified as chimeric reads, which may reveal artifactual or sheer chimeric RNAs (chRNAs) (see Discussion).

CRAC processes reads one by one, first by determining the location breaks, then analyzing the support profile, and applying the inference rules whenever possible. A read is classified according to the events (SNV, error, indels, splice, or chimera) that are predicted, and its mapping unicity or multiplicity. Additional file 1 gives an overview of the classification. The CRAC algorithm is described for the analysis of an individual read, but its output can be parsed to count how many reads led to the detection of the same SNV, indel, splice, or chimera; this can serve to further select candidates. CRAC accepts the FASTA and FASTQ formats as input, and outputs distinct files for each category, as well as a SAM formatted file with mapping results.

In describing CRAC's method above, we first assumed simplifying conditions: especially the absence of false locations (FLs) and border cases. Some details will clarify how the actual procedure handles real conditions.

#### Differences with the genome at a read's extremities (border cases)

Border cases are not processed with a specific procedure by CRAC; instead, the sequencing depth of NGS data indicates border cases. While processing a read, if an event (say, a splice junction) generates a break at one of the read's extremities, the coverage ensures that the same event is likely located in the middle of other reads, and will be detected when processing these. The border case read is classified either as undetermined or biologically undetermined depending on its support profile, and it is output in the corresponding files.

#### False locations (Figure 1d)

Our criterion to set *k *ensures a low average probability of a random *k*-mer match on the genome [19], but it does not prevent random matches, which we term false locations. Compared to true (unique or multiple) locations, FL of a *k*-mer will generally not be coherent with those of neighboring *k*-mers. It may also alter the break length in an unexpected manner, making the break length another criterion of verification (Rule 1). When a read matches the genome, CRAC considers ranges of *k*-mers having coherent locations to infer its true genomic position. In the case of a break, CRAC faces two difficulties. First, when a FL happens at the end of a break, CRAC may incorrectly delimit the break. When a FL occurs inside a break, it makes adjacent false breaks, termed mirage breaks. In both cases, the FL may cause CRAC to avoid Rule 1, apply Rule 2, and predict a false chimeric read. To handle a FL at a break end, CRAC uses a break verification procedure, and it applies a break fusion procedure to detect and remove mirage breaks.

These procedures are detailed in Additional file 2, which also includes explanations of the distinction of dropping and non-dropping supports around a break, on read mapping at multiple locations, on the subclassification of chimeric reads, and on the simulation protocol.

## Results

We evaluated CRAC for mapping reads, predicting candidate SNVs, indels, splice junctions, and chimeric junctions, and compared it to other tools. Simulated data are needed to compute exact sensitivity and accuracy levels, while real data enable us to study predictions with biologically validated RNAs. For simulating RNA-seq, we first altered a reference genome with random substitutions, indels, and translocations to derive a mutated genome, then reads were sequenced *in silico *using FluxSimulator [22], the annotated RefSeq transcripts, and a realistic distribution of random expression levels (Additional file 2). As read lengths will increase, we used two simulated datasets to assess different strategies: one (hs75) with a typical read length of 75, another (hs200) with reads of 200 nt representing the future.

### Mapping with current (75 nt) and future (200 nt) reads

Mapping, that is, the process of determining the location of origin of a read on a reference genome, provides critical information for RNA-seq analysis. Currently used mappers (Bowtie, BWA, SOAP2 and Bowtie2) compute the best continuous genome-read alignments up to a certain number of differences [9,11,12,23]. CRAC and GSNAP [14], also consider discontinuous alignments to search for the locations of reads spanning a splice junction: they can find both continuous and spliced alignments.

An overview of mapping results with 75 nt reads (Table 1) indicates a high level of precision, but strong differences in sensitivity among tools. All achieve a global precision >99%, meaning that output genomic positions are correct. Bowtie, BWA, and SOAP2 are similar by design, and all look for continuous alignments with a few substitutions and small indels. Although its approach differs, GASSST also targets these (and is better for longer indels). Even within this group, the sensitivity varies significantly: from 70% for GASSST to 79% for BWA. These figures are far from what can be achieved on RNA-seq data since GSNAP and CRAC, which also handle spliced reads, reach 94% sensitivity: a difference of at least 15 points compared to widely used mappers (Bowtie2 included). As only uniquely mapping reads were counted, the sensitivity cannot reach 100%: some reads are taken from repeated regions and thus cannot be found at a unique location.

**Table 1:** Comparative evaluation of mapping sensitivity and precision

	75 bp		200 bp	
Tool	Sensitivity	Precision	Sensitivity	Precision
Bowtie	75.42	99.59	55.72	**99.81**
Bowtie2	76.64	99.26	62.31	98.78
BWA/BWA-SW	79.29	99.13	68.66	96.86
CRAC	*94.51*	*99.72*	**95.9**	*99.79*
GASSST	70.73	99.09	59.43	97.86
GSNAP	**94.62**	**99.88**	*84.84*	99.28
SOAP2	77.6	99.52	56.08	99.78

We compared the sensitivity and precision of different tools on the human simulated RNA-seq (42M, 75 nt and 48M, 200 nt) against the human genome for mapping. The sensitivity is the percentage of correctly reported cases over all sequenced cases, while the precision is the percentage of correct cases among all reported cases. Values in bold in the three tables indicate the maximum of a column, and those in italics the second highest values. For all tasks with the current read length, CRAC combines good sensitivity and very good precision. Importantly, CRAC always improves sensitivity with longer reads, and delivers the best sensitivity while keeping a very high precision.

**Figure 2:**
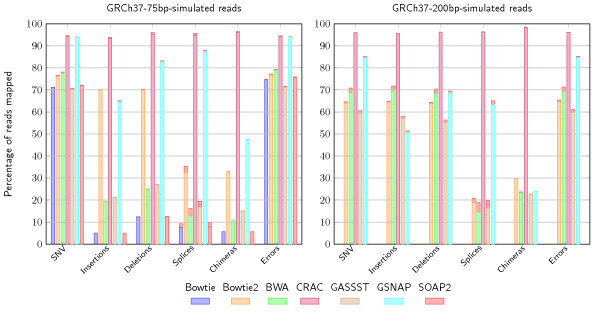
**Comparison of mapping results by category for seven tools**. The figure shows the mapping by event category on simulated RNA-seq against the human genome on datasets with short and long reads (left 42M, 75 nt; right 48M, 200 nt) for seven different mapping tools: Bowtie, Bowtie2, BWA/BWA-SW, CRAC, GASSST, GSNAP, and SOAP2. We consider six categories of reads depending on whether they contain an SNV, an insertion, a deletion, a junction, a sequence error, or a chimeric splice junction (a chimera). In each category, the bar is the percentage of those reads mapped at a unique location by the corresponding tool. The red tip at the top of the bar is the percentage of incorrectly mapped reads. With 75 nt reads, CRAC is better than the other tools, reaching a sensitivity >90% and a precision >95% whatever the category. The other tools except GSNAP are below 50% sensitivity for mapping reads in categories where spliced alignments are needed (for which they are not intended) and for reads containing insertions or deletions. With 200 nt reads, CRAC remains by far the most sensitive and specific tool; the difference between CRAC and GSNAP and Bowtie2 increased in all categories. Compared to short reads, the other tools had a better mapping of insertion and deletion containing reads. SNV: single nucleotide variant

One gets a clearer view by considering the subsets of reads that carry an SNV, an indel, an error, a splice, or a chimeric junction (Figure 2). Strikingly, CRAC is the only tool that achieves similar performance, a sensitivity of 94% to 96%, in all categories. For instance with indels, GSNAP yields 65% and 83% sensitivity on insertions and deletions respectively, Bowtie2 yields 70% sensitvity for both insertions and deletions, while the other tools remain below 30%. BWA, GASSST, Bowtie, and SOAP2 output continuous alignments for 9% to 19% of spliced reads, and Bowtie2 up to 35%. Although their output locations are considered correct, for they are in one exon, their alignments are not. Such reads are considered as mapped and thus not reanalyzed by tools like TopHat or MapSplice in a search for splice junctions, which may lead to missing junctions.

Analyzing longer reads (200 nt) is another challenge: the probabilities for a read to carry one or several differences (compared to the reference) are higher. In this dataset, 36% of the reads cover a splice junction, and 50% carry an error. Compared to the 75 nt data, while their precision remains >99%, BWA, GASSST, Bowtie, Bowtie2, SOAP2, and GSNAP, have lower sensitivity (approximately 10 points less for BWA-SW, GASSST, and GSNAP, 14 for Bowtie2, and 20 for Bowtie). Only CRAC remains as precise and gains 1.5 points in sensitivity (Table 1). The detail by category confirms this situation (Figure 2), showing CRAC is better than current tools. CRAC's *k*-mer profiling approach can accurately handle reads altered by distinct categories of biological events, and importantly adapts well to longer reads.

The same analyses have been performed on *Drosophila *datasets and these show that all tools perform better, but the differences between tools remain (Additional file 3). The run times and memory usage of all tools are given in Additional file 3, Table S3. CRAC requires a large memory and its run time for analyzing reads ranges between that of Bowtie and TopHat, which are practical tools. Indexing the human genome with crac-index takes two hours on an x86_64 Linux server on a single thread and uses 4.5 gigabytes of memory.

### Predicting distinct categories of biological events

Mapping is not a goal per se, but only a step in the analysis; the goal of read analysis is to detect candidate biological events of distinct categories (SNVs, indels, and splice and chimeric junctions) from the reads. The question is: if, for example, there is an SNV or splice junction that has been sequenced, can it be predicted and not buried under a multitude of false positives (FPs)? Here, sensitivity and precision are relative to the number of events, not to the number of reads covering them. We assessed CRAC's prediction ability and compared it to splice junction prediction tools on our simulated datasets.

**Figure 3:**
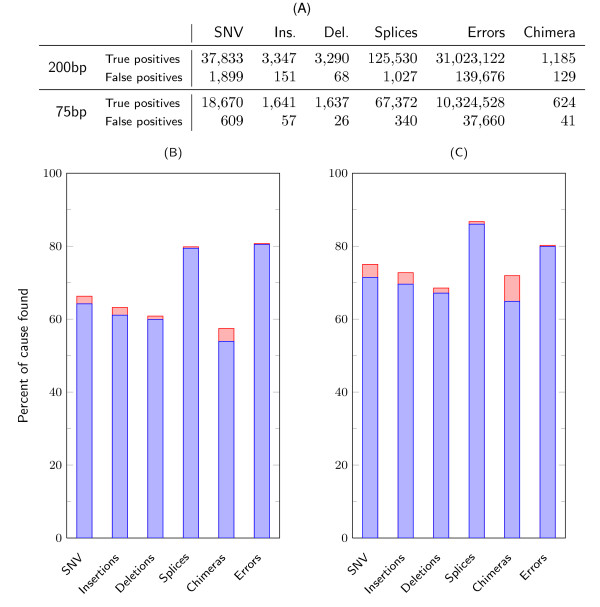
**Sensitivity and precision of CRAC predictions by category for human simulated data**. **(A) **Absolute numbers of true and false positives reported by CRAC. These figures are the number of distinct events, say SNVs, reported by CRAC, not the number of reads containing the same SNV. False positives represent a small fraction of its output, thereby indicating a high level of precision. **(B) **and **(C) **For each category, the figure shows the proportion of events found by CRAC for the 75 nt and 200 nt datasets. The blue bars are the true positives, while the red bars on top are the false positives. The height of a blue bar gives CRAC's sensitivity, and the relative height of the red part of the bar gives the precision. For the two read lengths, for all categories the sensitivity increases with longer reads, while the precision in each category varies only a little. SNV: single nucleotide variant

Figure 3 gives CRAC's precision and sensitivity for each category of events and for sequencing error detection. For SNVs and indels (<15 nt), CRAC achieves a sensitivity in the range [60,65]% and a precision in the range [96.5,98.5]% (Figure 3), making it a robust solution for such purposes. Typically, CRAC missed SNVs that either have low coverage (42% of them appear in ≤2 reads) or are in reads carrying several events (66% of missed SNV reads also cover a splice junction). For the splice junction category, CRAC delivers 340 false and 67,372 true positives (TPs).

An overview and the effect of read length on sensitivity and precision are shown in Table 2. With 75 nt, all splice detection tools achieve good sensitivity, ranging from 79% for CRAC to 85% for TopHat, but their precision varies by more than 10 points (range [89.59,99.5]). CRAC reaches 99.5% precision and thus outputs only 0.5% FPs; for comparison, MapSplice and GSNAP output four times as many FPs (2.32% and 2.97%), while TopHat yields 20 times more FPs (10.41%). With 200 nt reads, tools based on *k*-mer matching, that is CRAC and MapSplice, improve their sensitivity (6.5 and 5 points respectively), while mapping-based approaches (GSNAP and TopHat) lose, respectively, 12 and 30 points in sensitivity, and TopHat2 gains 6.4 points in sensitivity. With long reads, CRAC has the second best sensitivity and the best precision (>99%). It also exhibits a better capacity than MapSplice to detect junctions covered by few reads: 15,357 vs 13,101 correct junctions sequenced in ≤4 reads.

**Table 2:** Comparative evaluation of splice junction prediction tools

	75 bp		200 bp	
Tool	Sensitivity	Precision	Sensitivity	Precision
CRAC	79.43	**99.5**	*86.02*	**99.18**
GSNAP	*84.17*	97.03	72.94	97.09
MapSplice	79.89	*97.68*	84.72	*98.82*
TopHat	**84.96**	89.59	54.07	94.69
TopHat2	82.25	92.71	**88.65**	91.35

We compared the sensitivity and precision of different tools on the human simulated RNA-seq (42M, 75 nt and 48M, 200 nt) against the human genome for splice junction prediction. The sensitivity is the percentage of correctly reported cases over all sequenced cases, while the precision is the percentage of correct cases among all reported cases. Values in bold in the three tables indicate the maximum of a column, and those in italics the second highest values. For all tasks with the current read length, CRAC combines good sensitivity and very good precision. Importantly, CRAC always improves sensitivity with longer reads, and yields the best precision (that is the fewer false positives) over all solutions, even against specialized tools like TopHat.

A comparison using chimeric RNAs shows that CRAC already has an acceptable balance between sensitivity and precision with 75 nt reads (53% and 93%, respectively), while the sensitivities of TopHat-fusion and MapSplice remain below 32% (Table 3). With 200 nt reads, only CRAC is able to predict chimeric splice junctions with acceptable precision, and sensitivity is improved compared to shorter reads (Table 3 and Additional file 3).

**Table 3:** Comparative evaluation of chimeric RNA prediction tools

	75 bp		200 bp	
Tool	Sensitivity	Precision	Sensitivity	Precision
CRAC	*53.89*	*93.84*	*64.86*	**90.18**
MapSplice	2.33	0	2.63	0.01
TopHat2	**77.72**	7.32	**70.72**	*12.50*
TopHat-fusion	32.73	42.02		
TopHat-fusion-post	12.26	**97.22**		

We compared the sensitivity and precision of different tools on the human simulated RNA-seq (42M, 75 nt and 48M, 200 nt) against the human genome for chimeric junction prediction. The sensitivity is the percentage of correctly reported cases over all sequenced cases, while the precision is the percentage of correct cases among all reported cases. Values in bold in the three tables indicate the maximum of a column, and those in italics the second highest values. For all tasks with the current read length, CRAC combines good sensitivity and very good precision. Importantly, CRAC always improves sensitivity with longer reads, and has the best balance between sensitivity and precision. TopHat-fusion could not process 200 nt reads.

As with mapping, for all categories of event, CRAC's prediction performance improves with longer reads (Figure 3).

### Predicting distinct categories of biological events on real data

#### Splice junction prediction

To evaluate CRAC's ability to detect splice junctions in real RNA-seq data, we compared it to state-of-the-art tools (TopHat, GSNAP, and MapSplice) on a dataset of 75 million stranded 100 nt reads (ERR030856; see Additional file 4 Table S1). Splice junctions were searched for using each tool and then compared to human RefSeq transcripts. Each found junction consists of a pair of genomic positions (that is, the exons 3' end and 5' start) and we considered that it matches a RefSeq junction if the positions were equal within a 3 nt tolerance. Found junctions were partitioned into *known*, *new*, and *other junctions *(KJs, NJs, and OJs, respectively). Known junctions are those already seen in a RefSeq RNA, new ones involve RefSeq exons but in a combination that has not yet been observed in RefSeq, while the remaining junctions go into the class other. Note that known RefSeq junctions include both junctions between neighboring exons and alternative splicing cases, mostly caused by exon skipping or alternative splice sites [24]. Novel junctions will provide new alternative splicing candidates, while junctions in class other are totally new candidate RNAs.

**Figure 4:**
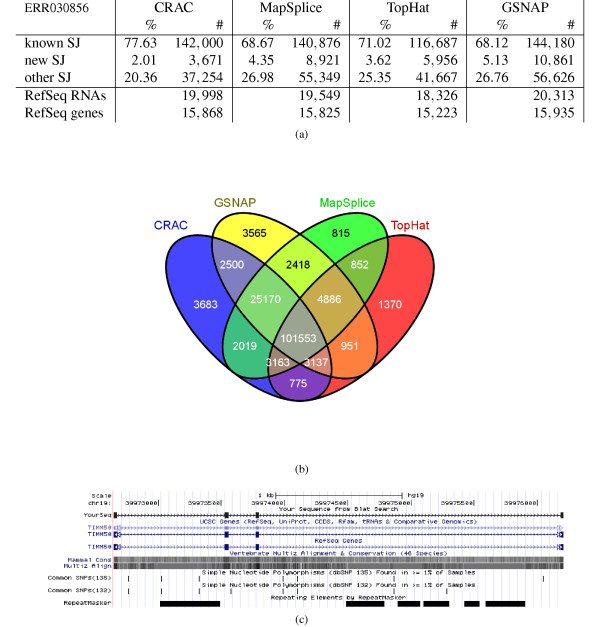
**Splice junction detection using human real RNA-seq: comparison and agreement**. The figure shows the detection of splice junctions by CRAC, MapSplice, TopHat, and GSNAP for a human six-tissue RNA-seq library of 75M 100 nt reads (ERR030856). **(a) **Number and percentage of known, new, and other splice junctions detected by each tool with +/−3 nt tolerance for ERR030856. **(b) **Venn diagram showing the agreement among the tools on known RefSeq splice junctions (KJs). Additional file 4 has pending data for novel junctions (NJs) and RefSeq transcripts. **(c) **A read spanning four exons (2 to 5) and three splice junctions of the human TIMM50 gene displayed by the UCSC genome browser. The included exons, numbers 3 and 4, measure 32 and 22 nt, respectively. So exon 3 has exactly the *k*-mer size used in this experiment. KJ: known splice junction; SJ: splice junction

For each tool, the distribution of junctions in the classes, and the number of detected RefSeq RNAs and genes (those having at least one KJ or NJ) are given in Figure 4a. The agreement on known junctions (KJs) among the tools is shown as a Venn diagram (Figure 4b); see Additional file 4 for the corresponding figures and a Venn diagram on novel junctions (NJs). Clearly, MapSplice, GSNAP, and CRAC find between [140,876;144,180] known junctions and all three agree on 126,723 of them. GSNAP and CRAC share 93% of CRAC's reported known junctions. TopHat reports about 25,000 junctions fewer than the other tools, and only 1,370 of its junctions are not detected by any of them. For instance, CRAC covers 93% of TopHat's KJs. As known junctions likely contain truly expressed junctions of well-studied transcripts, these figures assess the sensitivity of each tool and suggest that in this respect CRAC equals state-of-the-art tools. Logically, the numbers vary more and the agreements are less pronounced among novel junctions. A marked difference appears within the class other: CRAC yields only 20.36% of other junctions, while with the other tools find [25;27]% of detected junctions.

To further test CRAC with negative controls, we created a set of 100,000 random junctions by randomly associating two human RefSeq exons, and for each we built a 76 nt read with the junction point in the middle of the read (see Additional file 4). These 100,000 reads were processed by CRAC with *k *= 22 and it predicted no splice junctions.

Are the junctions in classes New and Other interesting candidates? To check predicted junctions, we extracted a 50 nt sequence around each inferred junction point and aligned it with BLAST against the set of human mRNAs/ESTs (for details and results see Additional file 4). A 50 nt sequence can either match over its entire length on an EST or match only one side of the junction but not both exons. The former confirms the existence of that junction in the ESTs and yields a very low E-value (≤10^-15^), while the latter has a larger value (≥10^-10^). As expected, at least 95% of KJs have very low E-values against ESTs, whatever the tool. Among new and other junctions, BLAST reports good alignments for respectively 68% and 69% of CRAC's junctions. The corresponding figures are 47% and 47% for GSNAP, 49% and 50% for MapSplice, 51% and 44% for TopHat. The percentages of OJs and NJs confirmed by mRNAs are >13% for CRAC and <8% for all other tools (excepted for OJs with TopHat, which was 17%, the same as CRAC). If we consider all junctions, 93% of CRAC junctions align entirely to an EST with a good hit. Whatever the class of the junctions, CRAC predicts more unreported junctions that are confirmed by mRNAs or ESTs than the other tools. This corroborates the precision rates obtained by these tools on simulated data.

Regarding expressed transcripts, all tools detect >18,000 transcripts and agree on 17,131 of them (Additional file 4 Figure S1). GSNAP and CRAC agree on 97% (19,431) of CRAC's detected transcripts, expressed in 15,589 distinct genes, which represents 87% of the 17,843 multi-exon RefSeq genes.

By simultaneously exploiting the genomic locations and support of all *k*-mers gives CRAC some specific abilities for junction detection. CRAC reports 752 junctions with an intron larger than 100 knt. The other tools find fewer of these junctions: 695, 589, and 470 for GSNAP, MapSplice, and TopHat, respectively, but both MapSplice and TopHat find fewer than expected by chance according to the global agreement between these tools (Additional file 4). CRAC also reveals 69,674 reads that cover exactly two known RefSeq junctions, that is, that cover three distinct exons and include one of them. An example of a double junction covering a 29 nt exon of the CALM2 gene is shown in Additional file 4. Moreover, of 9,817 of such junctions, GSNAP, MapSplice, and TopHat, find respectively 8,338, 9,167, and 7,496, which for GSNAP and TopHat is less than expected by taking a random sample of junctions (Additional file 4). CRAC even maps reads spanning 3 successive junctions (4 exons), and finds an additional 89 junctions, which are not all reported by current tools. For instance, GSNAP does not map such reads. An example for the TIMM50 gene is shown in Figure 4c. Altogether, these results suggest that numerous new splice junctions, even between known exons, remain to be discovered [25], but other predicted junctions that would correspond to completely new transcripts may also be due in part to the inaccuracy of splice junction prediction tools. In this respect, CRAC seems to ally sensitivity and precision, which should help discriminate true from false candidates, while it has good potential for detecting multiple junctions occurring within the same read. Such reads with multiple junctions will be more abundant with longer reads, and are useful for the reconstruction of transcripts, which is done on the basis of detected junctions [26].

#### Comparisons of chimeric splice junction prediction

Edgren et al. used deep RNA-sequencing to study chimeric gene fusions in 4 breast cancer cell lines (BT-474, KPL-4, MCF-7, and SK-BR-3; see Additional file 4 Table S1); they found 3 known cases and validated 24 novel intergenic fusion candidates (that is, involving 2 different genes) [27]. As CRAC, TopHat-fusion can predict both intragenic and intergenic chRNA candidates and identify a chimeric junction in a spanning read [16]. For evaluation purposes, we processed each library with TopHat-fusion and CRAC, and compared their results. TopHat-fusion exploits both the read sequence and the read pairs, while CRAC uses only the single read sequence. Otherwise, TopHat-fusion per se^1 ^and CRAC both select potential chRNAs based on computational criteria. We further filtered out all candidate chimeric reads for which an alternative, colinear alignment was found by GSNAP (Additional file 4). Then, filtered predictions were compared with valid chRNAs. A post-filtering script, called TopHat-fusion-post, based on biological knowledge, can be applied to TopHat-fusion results, but in [16] its parameters were chosen 'using the known valid fusions as control', and may have biased the comparison. So, we recalculated all predictions using TopHat-fusion with and without TopHat-fusion-post.

The numbers of distinct candidate chimeric junctions (chRNA for short) and chimeric single reads detected by both tools in each library are given in Table 4.

**Table 4:** Chimeric RNA detection in breast cancer libraries

Edgren libraries	CRAC				TopHat-fusion			
	Raw		After GSNAP		Raw		After GSNAP	
	Number of chRNAs	Number of reads	Number of chRNAs	Number of reads	Number of chRNAs	Number of reads	Number of chRNAs	Number of reads
BT-474	692	9,661	153	460	109,711	349,801	81,327	189,523
KPL-4	407	5,157	60	199	32,412	98,330	23,075	53,165
MCF-7	466	3,475	90	180	42,738	121,544	27,267	57,676
SK-BR-3	703	9,354	152	577	86,249	241,219	61,494	130,682

TopHat-fusion reports approximately 200 times more raw candidates than CRAC; this ratio increases after filtering. Comparison with the set of validated chRNAs by Edgren et al. [27] shows that both the filtered and unfiltered predictions of CRAC and TopHat-fusion include respectively 20 and 21 true chRNAs and they agree for 17 of them.

The 50 nt reads, which are well suited for Bowtie and TopHat, are unfavorable for CRAC, which performs better with longer reads. Globally after filtering with GSNAP, TopHat-fusion reports a total of 193,163 chRNAs, while CRAC outputs 455: a 600-fold difference. Compared to the results obtained above for a six-tissue library (ERR030856), TopHat-fusion reports about as many chimeric junctions as CRAC, GSNAP, or MapSplice for normal splice junctions. Such a set likely includes a majority of false positives as already noted [16], and cannot help in estimating the quantity of non-colinear RNAs in a transcriptome. In comparison, CRAC's output is a practical size and allows an in-depth, context-dependent investigation for promising candidates for validation.

In CRAC's output, intragenic and intergenic chRNAs account for 58% and 42% respectively, and are partitioned into five subclasses (Methods, Additional file 5). Looking at the intersection, TopHat-fusion also outputs 76% (346) of the chRNAs found by CRAC, therefore providing additional evidence in favor of their existence, since the presence of some supporting read pairs is a mandatory criterion in TopHat-fusion [16] (Additional file 5).

When compared with the set of validated chimeras of Edgren et al. [27], TopHat-fusion and CRAC detected 21 and 20 out of 27, and agreed on 17 of them (Table 5).^2 ^The first 20 cases were found by CRAC, and the 7 remaining ones were not predicted by CRAC; however, for the final 2, we could not detect any read matching the 15 to 20 nt over the junction. Among the seven cases CRAC misses, only one (BCAS4-BCAS3) is a false negative, four are uncertain with not enough expressed candidates (CPNE1-P13, STARD3-DOK5, WDR67-ZNF704, and PPP1R12A-SEPT10), and no read seems to match the junction of the two remaining ones (DHX35-ITCH and NFS1-PREX1). As the BCAS4-BCAS3 junction includes a substitution near the splice site, the reads carry two events (SNV plus junction): CRAC does not exactly position the junction and outputs them in the BioUndetermined file, whose exploration could extract BCAS4-BCAS3 as a candidate (future work). For the four uncertain cases, the *k*-mer support over the junction break equals one, meaning that only one read matches the junction exactly; hence CRAC identifies a chimeric junction, but classifies them as uncertain candidates (Undetermined file). Three out of four are nevertheless detected by TopHat-fusion, but with two or one spanning reads (2,1,1) and few supporting pairs (6,5,0), thereby corroborating CRAC's view and confirming these are expressed at very low levels in this dataset.

**Table 5:** CRAC and TopHat-fusion predictions for the set of validated chimeric junctions from breast cancer libraries

Library	Fused genes	Chromosomes	5' position	5' strand	3' position	3' strand	Average support^a^	CRAC^b^	TopHat-fusion^c^
BT-474	SNF8-RPS6KB1	17-17	47,021,337	1	57,970,686	-1	36	Yes	Yes
BT-474	CMTM7-GLB1	3-3	32,483,329	-1	33,055,545	1	2	Yes	Yes
BT-474	SKA2-MYO19	17-17	57,232,490	-1	34,863,351	-1	6	Yes	Yes
BT-474	ZMYND8-CEP250	20-20	45,852,968	-1	34,078,459	1	9	Yes	Yes
BT-474	VAPB-IKZF3	20-17	56,964,572	1	37,934,021	-1	6	Yes	Yes
BT-474	ACACA-STAC2	17-17	35,479,452	-1	37,374,427	-1	46	Yes	Yes
BT-474	DIDO1-TTI1	20-20	61569147	-1	36,634,800	-1	2	Yes	Yes
BT-474	RAB22A-MYO9B	20-19	56,886,178	1	17,256,205	1	9	Yes	Yes
BT-474	MCF2L-LAMP1	13-13	11,371,8616	-1	113,951,811	-1	2	Yes	No
KPL-4	NOTCH1-NUP214	9-9	139,438,475	-1	134,062,675	1	2	Yes	Yes
KPL-4	BSG-NFIX	19-19	580,782	1	13,135,832	1	9	Yes	Yes
MCF-7	RPS6KB1-TMEM49	17-17	57,992,064	1	57,917,126	1	5	Yes	Yes
MCF-7	ARFGEF2-SULF2	20-20	47,538,548	1	46,365,686	-1	10	Yes	Yes
SK-BR-3	PKIA-RARA	8-17	79,485,042	-1	38,465,537	-1	7	Yes	Yes
SK-BR-3	TATDN1-GSDMB	8-17	125,551,264	-1	38,066,177	-1	334	Yes	Yes
SK-BR-3	KCNB1-CSE1L	20-20	47,956,856	-1	47,688,990	-1	6	Yes	No
SK-BR-3	CYTH1-EIF3H	17-8	76,778,283	-1	117,768,258	-1	11	Yes	Yes
SK-BR-3	SUMF1-LRRFIP2	3-3	4,418,012	-1	37,170,640	-1	4	Yes	Yes
SK-BR-3	SETD3-CCDC85C	14-14	99,880,273	1	100,002,353	1	3	Yes	No
SK-BR-3	PCDH1-ANKHD1	5-5	141,234,002	1	139,825,559	-1	2	Yes	Yes
BT-474	CPNE1-P13	20-20	34,243,123	NA	43,804,501	NA	1	No	Yes
BT-474	STARD3-DOK5	17-17	37,793,479	NA	53,259,992	NA	1	No	Yes
SK-BR-3	WDR67-ZNF704	8-8	124,096,577	NA	81,733,851	NA	1	No	Yes
MCF-7	BCAS4-BCAS3	20-17	49,411,707	NA	59,445,685	NA	3	No	Yes
KPL-4	PPP1R12A-SEPT10	12-2	80,211,173	NA	11,034,3414	NA	1	No	No
SK-BR-3	DHX35-ITCH	20-20	Unknown	NA	Unknown	NA	NA	No	No
SK-BR-3	NFS1-PREX1	20-20	Unknown	NA	Unknown	NA	NA	No	No

NA: not applicable

^a ^Average support value over the junction *k*-mers

^b ^Detected by CRAC

^c ^Detected by TopHat-fusion

CRAC and TopHat-fusion predictions on the set of validated chimeric junctions from four breast cancer libraries [27]. The first 20 cases were found by CRAC, and the 7 remaining ones were not predicted by CRAC; however, for the final 2, we could not detect any read matching the 15 to 20 nt over the junction. A short read length penalizes CRAC: indeed, with *k *= 22, only the 6 (= 50 - 2 × 22) middle positions of a read could be used to locate any event (splices or mutations) exactly. Hence we expect that the spanning reads by which a chRNA is amenable to detection by CRAC to be rare. NA: not applicable. Columns: library, fused genes ID, annotation of the junction points, chromosomes, 5' position and strand, 3' position and strand, average support value over the junction k-mers, detection by CRAC and by TopHat-fusion (THF).

Considering validated intergenic chRNAs [27], the sensitivity over the 27 valid chRNAs is comparable between TopHat-fusion (77% = 21/27) and CRAC (74% = 20/27), while the precision over the total number of candidates is markedly in favor of CRAC (21/143,003≃0.01%vs20/192≃10.4%;^3 ^Table 5, Additional file 5). Clearly, some experimentally validated chRNAs (like DHX35-ITCH or NFS1-PREX1), happen to have no read spanning their junction, and thus should not be computationally predicted as candidates on the basis of this read data. This important statement illustrates how difficult computational chRNA prediction is, thereby emphasizing the quality of CRAC's analysis. Moreover, the evidence suggests that other promising candidate chRNAs populate CRAC's results.

Numerous chRNAs are predicted in classes 3/5, where the RNA non-colinearity appears as an inversion. CRAC detects three such chRNAs within the MAN1A2 gene, which recur in up to three out of four breast cancer libraries, and in a K562 library. These specific inversions in MAN1A2 are described as post-transcriptional exon-shuffling RNAs and found highly expressed in several acute lymphoblastic leukemia samples [28]. Our results support the existence of such mRNA-exhibiting shuffled exons, as well as cases where the inversion is short, sometimes inducing a repeat within the read (see an example in the LONP1 gene given in Additional file 4).

Notably, among 455 chRNAs, CRAC reports 36 chRNAs that appear to recur in two, three, or even all four breast cancer libraries (Additional file 5). Among these 36 chRNAs: 24 are intra- and 12 are inter-chromosomal, 20 are intragenic, while 16 fuse different genes. Moreover, 35 out of 36 (including the MAN1A2 and LONP1 cases) harbor exactly the same junction point in all libraries in which they were detected. Previous investigations of these libraries [16,27] did not report any recurrent chRNAs. However, when we ran TopHat-fusion, it also output 23 of these chRNAs among 193,163 candidates.

For instance, we found a HSPD1-PNPLA4 chRNA in both KPL-4 and SK-BR-3 libraries: PNPLA4 (GS2) is highly expressed in human SW872 liposarcoma cells [29], while HSPD1, the heat shock protein Hsp60, shows a broad antiapoptotic function in cancer [30]. Among the intragenic chRNAs, we observed in all four libraries a non-colinear chRNA within GNAS, a gene coding for the G-protein alpha subunit, which is known to be associated with multiple human diseases including some cancers [31], and was recently found to be recurrently mutated in cystic pancreatic lesions related to invasive adenocarcinomas [32], as well as amplified in breast cancers [33]. Moreover, we also found the same CTDSPL2-HNRNPM chimeric RNA in the BT-474, MCF-7, and SK-BR-3 libraries. Both genes belong to the heterogeneous nuclear ribonucleoprotein family and play a pivotal role in pre-mRNA processing. Importantly, HNRNPM regulates the alternative splicing of carcinoembryonic antigen-related cell adhesion molecule-1 (CEACAM1) in breast cancer cells [34].

## Discussion

CRAC is a multi-purpose tool for analyzing RNA-seq data. In a single run it can predict sequencing errors, small mutations, and normal and chimeric splice junctions (collectively termed events). CRAC is not a pipeline, but a single program that can replace a combination of Bowtie, SAMtools, and TopHat/TopHat-fusion, and can be viewed as an effort to simplify NGS analysis. CRAC is not simply a mapper, since it uses local coverage information (in the support profile) before computing the genomic position of a read. In contrast to the current paradigm, mapping and post inferences are not disjoint steps in CRAC. Instead, it implements a novel, integrated approach that draws inferences by simultaneously analyzing both the genomic locations and the support of all *k*-mers along the read. The support of a *k*-mer, defined as the number of reads sharing it, approximates the local read coverage without having the reads mapped. The combined *k*-mers location and support profiles enable CRAC to infer precisely the read and genomic positions of an event, its structure, as well as to distinguish errors from biological events. Integration is not only the key to an accurate classification of reads (Additional file 1), but it avoids information loss and saves re-computation, and is thereby crucial for efficiency. Indeed, CRAC takes more time than state-of-the-art mappers, but is considerably faster than splice junction prediction tools (for example, Bowtie plus TopHat). The other key to efficiency is the double-indexing strategy: a classical FM-index (where FM stands for Ferragina - Manzini) for the genome and the Gk arrays for the reads [21]. This makes CRAC's memory requirement higher than that of other tools, but fortunately computers equipped with 64 gigabytes of memory are widespread nowadays. Experiments conducted on simulated data (where all answers are known), which are necessary for assessing a method's sensitivity, have shown that for each type of prediction CRAC is at least competitive or surpasses current tools in terms of sensitivity, while it generally achieves better precision. Moreover, CRAC's performances further improve when processing longer reads: for example on 200 nt reads, it has 85% sensitivity and 99.3% precision for predicting splice junctions.

CRAC analyzes how the location and support profiles vary and concord along the read. Hence *k*-mers serve as seeds (in the genome and in the read set), and *k *is thus a key parameter. Its choice depends on the genome length [19], and quite conservative values - *k *= 22 for the human genome - have been used in our experiments. Smaller *k *values are possible with smaller genomes (like bacterial ones). *k *affects the number of false genomic locations (FLs) that occur in the profile; a FL indicates a wrong location for a *k*-mer, which differs from the location of origin of the sequenced molecule. This tends to induce a false location for the read (mapping) or a false location for a junction border (normal and chimeric junction prediction). However, CRAC uses two criteria to avoid these pitfalls: the coherence of locations for adjacent *k*-mers over a range and the concordance of locations for the *k*-mers around the break (especially in the break verification and fusion procedures; see Additional File 2). When *k*-mers surrounding the break have a few, but several, locations, CRAC examines all possible combinations, and as FL occurrences are governed mainly by randomness, this eliminates discordant positions. FLs have a larger effect on the prediction of chimeras. Overall, the results on both simulated and real data, like the improved mapping sensitivity (+15 points compared to Bowtie, BWA, and SOAP2), show that CRAC makes accurate predictions with conservative values. *k *controls the balance between sensitivity (shorter seeds) and precision. The breast cancer libraries we used have 50 nt reads, but CRAC could still find 74% of the chimeric RNAs validated by Edgren et al. [27]. Of course, the *k *value has two limitations: first, the minimal exon size detectable in a read is ≥*k*, second, reads must be long enough (>40 nt with *k *= 20 for the human genome). However, NGS is progressing towards longer reads, which should become standard, and Figure 4c illustrates well CRAC's ability to detect short exons within single reads. The *k*-mer profiling approach detects events located near the read extremities, but cannot exactly determine their position in the read. Hence the inference rules cannot be fully applied, and CRAC classifies such reads as incompletely determined (Undetermined and BioUndetermined files). However, the position of an event in a read is random, and thus, the high coverage delivered by NGS nearly ensures that the same event occurs in the middle of other reads covering it. Consequently, *border cases *do not hinder CRAC from detecting mutations, splice junctions, etc. Only errors escape this rule since they are mostly read specific. A more complex drawback of *k*-mer profiling is when two events are located <*k *positions apart on the genome (see the BCAS4-BCAS3 chimera); again such cases even with a high support are not fully resolved and end up in the BioUndetermined file. A post-processing of reads in this file, for example by an alignment program, could clearly save such cases. Obviously, such cases are rare, and we keep this as future work. As briefly mentioned, *k*-mer profiling also detects when reads span a repeat border region, which should help in inferring the locations of mobile genetic elements, duplications, or copy number variations; this suggests further developments and CRAC's usefulness for analyzing genomic data.

Determining the correct genomic location of reads is crucial information for any NGS data analysis and especially for cataloging all transcripts of a cell with RNA-seq. Generally, a mapping step computes this information using efficient, well-known tools (BWA, Bowtie, and SOAP2), but the mapping sensitivity is rarely questioned. We performed extensive mapping tests on simulated data, which showed that sensitivity can truly be improved and that CRAC makes a significant step in this direction. Of course by considering discontinuous alignments (as do CRAC and GSNAP) many reads covering splice junctions can be mapped, which BWA, Bowtie/Bowtie2, and SOAP2 cannot detect. However, the mapping results for categories of reads carrying one mutation, a short indel, or even errors indicate that classical mappers missed between 15 to 20 points in sensitivity, thereby confirming that the difference due to splice junction reads is critical even for other events, while CRAC performs equally well (>90%) whatever the category (Figure 2). The other way around, those tools are able to map 10% to 35% of reads containing a splice junction. This can negatively affect downstream analyses depending on the type of events under investigation. For instance to predict splice junctions, in the current strategy (TopHat, MapSplice, or TopHat-fusion), reads are first mapped with Bowtie to divide the collection into: (a) reads having a continuous alignment on the genome and (b) unmapped reads. The former serve further to delimit exons, and the latter are then processed again to search for spliced alignments. If a read that requires a discontinuous alignment is mapped by Bowtie, it is not considered by TopHat, MapSplice, or TopHat-fusion as potentially containing a junction, and they will not find a spliced alignment for it. In contrast, CRAC's *k*-mer profiling approach is flexible, reliable in this respect (Figure 3), and importantly, adapts well to longer reads (for example, 200 nt). This last point is key since longer reads will be available soon. They will much more likely incorporate not one, but several events - errors, mutations, splice junctions, etc. - and thus be harder to map. Whatever the class of required predictions, CRAC's sensitivity is always improved with longer reads. This is crucial for detecting multiple exons within single reads, and CRAC exhibits a better ability in this as exemplified by a transcript of TIMM50 gene (Figure 4c).

An issue in transcriptomics is to reliably extract the complete set of splice junctions with a minimal number of false positives [24]. In this regard, our results (Table 2) demonstrate that *k*-mer profiling approaches (MapSplice and CRAC) profit greatly in sensitivity from longer reads, and that CRAC is the tool with the highest precision whatever the read length. They also indicate that CRAC handles difficult cases with higher sensitivity, like long-distance splices, multi-exon reads, or RNA expressed at a low level. The analysis of a multi-tissue library shows that CRAC, GSNAP, and MapSplice have a very large (>90%) agreement on the set of reported known junctions (>140,000 distinct junctions), RefSeq transcripts, and genes, thereby providing evidence of their ability to extract splice junctions of well-annotated transcripts (Figure 4b and 4a). In contrast, TopHat misses 21% of these known RefSeq junctions. Comparatively, CRAC reports fewer novel or unknown junctions than other tools, and tends to be more conservative, which likely reflects its precision. Altogether, CRAC is a solution for exploring qualitatively the transcriptome of a sample with high sensitivity and precision, and thus provides the primary material for determining all transcript structures, which is indispensable for estimating the expression levels of all RNA isoforms [3,26].

Recent investigations have suggested that non-colinear RNAs are quantitatively more abundant in human transcriptomes than previously thought, underlining the structural diversity of these chimeric RNAs and their occurrence in cancers [8,27,28,35,36]. Predicting chimeric RNAs (chRNAs) is the most difficult and error-prone computation when analyzing RNA-seq. The combinatorial possibilities of aligning a read partly to two distinct regions on the same or different chromosomes [4] increase the likeliness of predicting FPs. It explains why filtering for suboptimal but colinear alignments of an apparent chimeric read may still help, and also partly why TopHat-fusion per se yields so many more chRNA candidates compared to CRAC (Table 4). Paired end reads are not sufficient: analyzing single reads by splitting them is inevitable for predicting the chimeric junction point; hence *k*-mer profiling also suits this purpose. Nevertheless, paired end reads are useful for performing a complementary consolidation of chRNA candidates, which we may develop in the future. However, chRNAs can occur at low expression levels and be much less expressed than their parental genes; this impels CRAC to rely less on the support profile than for mutation prediction. In addition, transcriptional noise or template switching during library preparation may generate true chimeric reads from biologically irrelevant chRNAs. Thus, subsequent criteria are definitely needed to prioritize chRNA candidates: the consistent finding of the same junction point has been suggested as an important one [27,36,37]. Notably, CRAC predicted for the four breast cancer libraries 36 recurrent chRNAs that were not reported previously [16,27], and 35/36 always harbor the same junction point in the different libraries and among the distinct reads predicting them. Several of these involve genes known to be implicated in tumorigenesis or tumor maintenance, like GNAS [31] or HSPD1 [30]. As CRAC outputs also included 74% of validated chRNAs with a single clear false negative, this shows that CRAC consistently reports interesting chRNA candidates based on the read data. As already mentioned, CRAC distinguishes between five chRNA classes, included those exhibiting small-scale sequence inversions, as illustrated by a chRNA within the LONP1 gene, which recurs in normal and tumoral libraries. We also reported cases of chRNAs, which although validated, do not constitute good candidates *for the computational inference step*, since not enough reads in the data support their existence. The latter point is critical and strengthens how difficult chimeric RNA prediction is.

Here, the *in silico *experiments focus on transcriptomic data, but the method is also applicable to genomic sequencing. For instance, the counterparts of splice junctions and chimeras in RNA-seq are large deletions and rearrangements (translocation, inversion, and displacement of a mobile element) in DNA. Thus, CRAC may also prove useful for genomic analyses.

## List of abbreviations

chRNA: chimeric RNA; EST: expressed sequence tag; FL: false location; indel: insertion or deletion; FP: false positive; KJ: known splice junction; NGS: next generation sequencing; NJ: new splice junction; nt: nucleotide; OJ: other splice junction; RNA-seq: RNA sequencing; SJ: splice junction; SNP: single nucleotide polymorphism; SNV: single nucleotide variant; TP: true positive.

## Competing interests

The authors declare that they have no competing interests.

## Authors' contributions

NP, MS and ER devised the algorithm. NP and MS developed the source code. All authors devised and analyzed the software comparisons and evaluations, as well as the analysis of real datasets. NP and MS performed the experiments. NP and MS prepared the figures. ER wrote the manuscript with contributions from all authors. ER and TC supervised the research. All authors read and approved the final manuscript.

## Endnotes

^a^ TopHat-fusion without the extra post-filtering script.

^b^ If TopHat-fusion-post is applied to TopHat-fusion's results with default parameters, it reports 27 chimera, 11 of them being validated chimeras, which is about half those reported by TopHat-fusion alone.

^c^ Only intergenic chRNAs are counted here.

## Supplementary Material

Additional file 1 **Figure with read classification performed by CRAC**. Click here for file

Additional file 2 **Additional description of the CRAC algorithm, the simulation of RNA-seq data, and the tools used for comparison**. Click here for file

Additional file 3 **Results for simulated RNA-seq data**. Click here for file

Additional file 4 **Results for real RNA-seq data**. Click here for file

Additional file 5 **Table with chimeric RNAs predicted for four breast cancer libraries**. Click here for file
