# Supplementary material for: CRAC: an integrated approach to the analysis of RNA-seq reads
Source: Genome Biol. 2013 Mar 28;14(3):R30. doi: 10.1186/gb-2013-14-3-r30 (PMC4053775; doi:10.1186/gb-2013-14-3-r30)
Supplement: Additional file 1 — Figure with read classification performed by CRAC. [file gb-2013-14-3-r30-S1.PDF]

CRAC: An integrated approach to analyse RNA-seq reads
Additional File 1
Overview of the read classification performed by CRAC.

Nicolas Philippe and Mikael Salson and Thérèse Commes and Eric Rivals

February 13, 2013

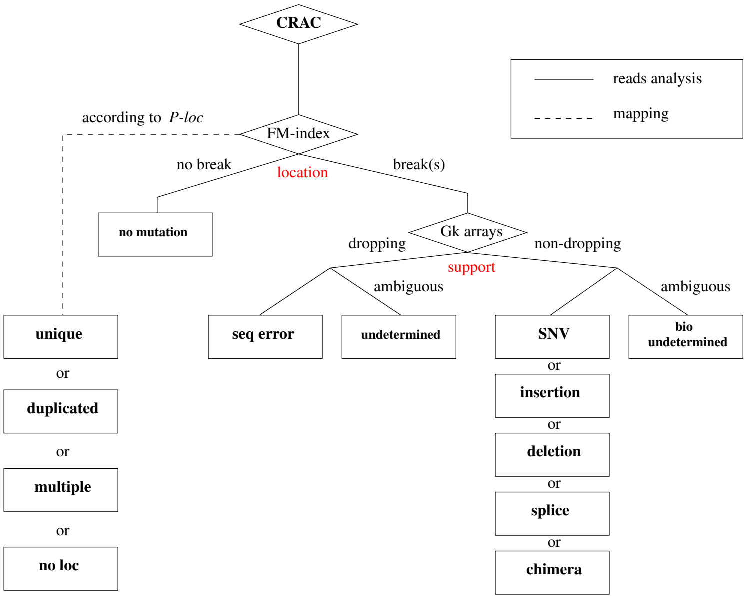

Figure 1: Overview of the read classification performed by CRAC. CRAC processes each read in turn and performs several predictions regarding its genomic locations, sequencing errors, the presence of mutations (SNV, insertion, and deletion), as well as normal or chimeric splice junctions. For each question the read can be assigned to one or several classes. Mapping: depending on the possible genomic locations of its k-mers obtained from the FM-index, CRAC decides whether the read has a unique (**unique**), or either a few (**duplicated**) or many genomic locations (**multiple**). When too many k-mers cannot be located the read is considered as having no location (**no loc**). Break: when the k-mer location profile contains a break, the Gk arrays are interrogated to analyze the support profile and decide whether it is due to a sequencing error or a biological event. In each case, the profiles may still be ambiguous and the read is then classified as not fully determined (**undetermined** or **bio undetermined**). Otherwise, according to the rules that distinguish different events the read is assigned to the relevant categories (**seq error**, **SNV**, **insertion**, **deletion**, **splice** or **chimera**).
