# Supplementary material for: CRAC: an integrated approach to the analysis of RNA-seq reads
Source: Genome Biol. 2013 Mar 28;14(3):R30. doi: 10.1186/gb-2013-14-3-r30 (PMC4053775; doi:10.1186/gb-2013-14-3-r30)
Supplement: Additional file 2 — Additional description of the CRAC algorithm, the simulation of RNA-seq data, and the tools used for comparison. [file gb-2013-14-3-r30-S2.PDF]

CRAC: an integrated approach to the analysis of RNA-seq reads
Additional File 2
Supplementary Methods: CRAC, simulation, and other tools.

Nicolas Philippe and Mikael Salson and Thérèse Commes and Eric Rivals

February 28, 2013

1 CRAC algorithm: details and real conditions

In the description of CRAC's method, we first assumed simplifying conditions; now we explain how the actual procedure deals with real conditions.

Differences with the genome at the read's extremities (border cases). This aspect does not influence CRAC's algorithm; rather, the sequencing depth of NGS data saves border cases. Indeed, the random fragmentation of molecules implies that a sequence difference will be uniformly located within the reads that cover these positions. The coverage ensures that these positions will likely be located in the middle of some other reads. Hence, if a biologicial event is missed in some border case read, it will be detected in other reads if the transcript is not rare. The results on simulated data illustrate well this situation. Reads with a difference near their extremities have a break lacking a start or an end (*i.e.* the location before or after the break cannot be determined): this prevents CRAC from identifying the type of difference and from finding its exact position in the read. As some characteristics of the difference are left undetermined, the read is classified either as "undetermined" or "biologically undetermined" depending on its support profile.

Genomic repeats Many reads are sequenced from repeated genomic regions. This translates in k-mers having multiple genomic locations. However, these locations are globally coherent. If a majority of located k-mers ($> 80\%$) are in this case, CRAC classifies reads as duplicated (≤ 5 matching locations) or repeated (> 5 locations). To apply Rule 1, CRAC needs to check the concordance of locations on each side of the break. When processing entirely or partly duplicated reads (not repeated ones), CRAC searches systematically each combination of locations and privileges coherent and concordant ones to reduce the risk of false inferences.

False locations (FL) Our criterion to set k ensures a low average probability of a random k-mer match on the genome [1], but it does not prevent random match, which we term false locations (FL). Compared to true (unique or multiple) locations, the FL of a k-mer will generally not be coherent with those of neighboring k-mers. It may also alter the break length in an unexpected manner: another criterion of verification (see Rule 1). When a read matches the genome, CRAC considers ranges of k-mers having coherent locations to infer its true genomic position. In case of a break in the location profile, CRAC faces two difficulties. First, when a FL happens at a border of a break, it may lead to an incorrect border. When a FL occurs inside a break, it makes up adjacent false breaks, termed *mirage breaks*. In both cases, if the FL is considered true, as it likely disagrees with the location of the other break border, it leads to avoid Rule 1, apply Rule 2, and to predict a false chimeric read. To handle FL at a break border, CRAC uses when necessary a *break verification* procedure, which checks the coherence of locations in the range $[j_b - \delta, j_b]$ (resp. $[j_a, j_a + \delta]$), the concordance of locations across the break, and the break length. This leads to discard the FL and identify the correct borders. Note that verifying the coherence of locations over δ consecutive k-mers is equivalent to considering a single $k+\delta$-mer and therefore diminishes the probability of FL. To detect and remove mirage breaks, CRAC applies a *break fusion* procedure. It checks the concordance of locations across each break, and also across a fused break, *i.e.* looking whether locations before the first break agree with those after the second break. This procedure, which handles duplicated locations by searching for all possible combinations, favors solutions with one or two breaks that avoids predicting chimeric reads.

Multiple differences When a read is affected by several differences (*i.e.* events *e.g.*, a substitution and a splice junction), two cases arise. Either these are k nucleotides apart, then two distinct breaks are found in the location profile (after verification and possible fusion to filter out false locations). Most of the time, k-mers locations across each break and across the two breaks are concordant, and CRAC detects two events. When the differences are too near ($< k$ nucleotides, which is less likely), a single break of unexpected length is found, and it hinders the precise inference of the types and positions of the differences (although the support analysis can still distinguish an error from biological event). Such reads are flagged as having a too large break, hiding several events, and are classified as "undetermined" or "biologically undetermined" depending on the support variation. A notable exception is the case of two substitutions, where we have $L = \ell > k$ and $\ell - k$ gives the distance between the two substitutions.

Rare splice junctions We mentioned that when the support is too low, the read is classified as "undetermined" because we cannot determine whether we are facing a sequencing error or a biological event. The situation is easier for splice junction, for it is unlikely that a sequencing error consists of a large insertion. Hence, when we detect a large insertion (given by the break type) with low support, we can confidently flag the read as being a "weak splice". This particularly highlights the need for integrating support analysis, with mapping and indel detection. Considering at once those informations enables CRAC to detect rare splices in a sample.

2 Simulation of RNA-seq data

To evaluate the sensitivity and precision of mapping programs and of tools for SNV, splice junction, or chimera predictions, we have produced benchmarks of simulated data for various genomes, read lengths, and amounts of sequencing. Using the program FluxSimulator, one can simulate RNA-sequencing from an annotated genome and control, through parameters, the length and number of reads, the amount of sequencing errors, and expression levels of annotated transcripts. However, this incorporates neither genetic mutations (substitutions and indels), nor translocations that make the sampled genome different from the reference genome used for mapping. Translocations are important for they occur in cancer cells and generate chimeric RNAs, which CRAC aims at predicting. To fill this gap, we developed a complementary tool to FluxSimulator called GenomeSimulator, which generates from the input reference genome, a mutated genome that is altered by random mutations and translocations. This program yields a mutated genome with modified annotations and the list of all alterations with their positions compared to the original reference. These files are stored and given to FluxSimulator, which then generates RNA-seq reads. Our system records all biological and erroneous differences compared to the reference, their exact positions on it and in the simulated reads, to allow the verification of mapping and prediction results.

Our goal is to compare several tools on their sensitivity and precision of predictions on RNA-seq data. With RNA-seq data, both events occuring at the genomic (single point mutations, indels, translocations) and at the transcriptomic levels (splicing events) are amenable to detection. Thus, to mirror real conditions our simulation protocol must incorporate the fact that the individual genome whose transcriptome is assayed differs from the reference genome the reads will be aligned to. The simulation procedure comprises two steps (Figure 1): 1/ a **genome alteration step**, in which the reference genome is randomly modified to account for individual genetic differences, 2/ a **RNA-seq simulation step**, where randomly chosen annotated genes are expressed and sequenced to yield reads. In the end, the simulation delivers a read collection and files recording the positions of genomic mutations, splice sites, chimeric junctions, in the reads and on the reference genome. One difficulty is to link the genomic alterations on the simulated reads in terms of positions on the reference genome.

To perform the RNA-seq simulation (step 2) we used the program FluxSimulator [2], which decomposes the simulation in gene expression, library construction and sequencing, and incorportates their systematic biases. However, for simulating an altered genome (step 1), we developed our own program that ensures an easy interconnection with FluxSimulator. Here, we provide a short overview of these computational simulation steps.

2.1 The genome simulation

This procedure modifies the sequence of the input reference genome by introducing random point mutations (SNV), insertions and deletions (or indels), as well as translocations. Substitutions and indels are introduced at random genomic locations at rates chosen by the user. By default, one every $1,000$ nucleotides will be substituted (a rate of 0.1%), while at $1/10,000$ positions, an indel will be introduced (a rate of 0.01%). At a substituted position, the new nucleotide is chosen at

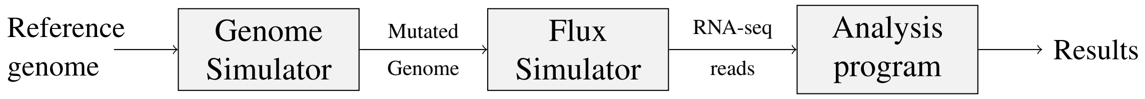

Figure 1: Overview of the simulation and analysis protocol. To generate a RNA-seq dataset, the Reference genome is first mutated by the Genome Simulator (step 1), the modified genome and annotations are input into the RNA-seq simulator (Flux Simulator step 2), which yields a collection of RNA-seq reads. This dataset is then analyzed by any desired tool (step 3). Its results can be confronted to the true errors, mutations, splice sites, chimeras, and its sensitivity and precision evaluated for each of these predictions.

random with equal probability among the three other possibilities. For indels, the length is chosen uniformly within a range $[1,15[$ nucleotides and the inserted sequence chosen randomly. Regarding translocations, whose goal is to generate gene fusions, the exchanged chromosomal locations are chosen within annotated genes. The genome simulator takes a gene annotation file as input to get the positions of all exons. For a translocation, two genes are chosen at random with an input probability, for each gene a breakpoint is chosen at random within its intronic regions, then we perform a bidirectional exchange of the start of one gene with that of the other gene, thereby creating two chimeric, or fusion genes. One starts with the sequence of gene 1 and ends with that of gene 2, and conversely. Exons are never splitted by this process, as we hypothesized that fusion genes with disrupted exons are counterselected by evolution. For the sake of simplicity, the simulator generates chimeras on the forward strand only. RNA-seq reads covering the fused genes will expressed chimeric RNAs; it is worth noting that both fused genes generated by a translocation are, as any other gene, not necessarily expressed, nor covered by reads.

2.2 The RNA sequencing simulation

FluxSimulator provides a RNA-seq simulation that includes all steps impacting the final reads: gene expression, library preparation, and sequencing. Besides the parameters, the input consists in a transcript annotation file in GFF format, which allows FluxSimulator to generate alternatively spliced RNAs for any single gene. We provided FluxSimulator with the RefSeq transcripts from the chosen species as extracted from Ensembl [3]. Another key parameter for testing read analysis tools is the sequencing error model. For substitutions, we used an error model issued from an analysis of the Illumina® sequencing technology with 75 nt reads [4] and we extrapolated this model for 200 nt long reads. In this model, indels are short (in the range $[1,5]$ nt) and their probabilities are much lower than that of substitutions. We also controlled the read length and the read numbers, and asked for single read sequencing only. We set up the parameters to obtain distributions of expression levels similar to that of real experiments [5] (see the RPKM graph for the Human datasets in Figure 2).

Detailed explanations on FluxSimulator parameters are available at http://fluxcapacitor.wikidot.com/simulator.

Sammeth, M., Lacroix, V., Ribeca, P., Guigó, R. The FLUX Simulator. http://flux.sammeth.

Name	Species	Number of reads in million	Read length in nt
hs-75	Human	42	75
hs-200	Human	48	200
dm-75	Drosophila melanogaster	45	75
dm-200	Drosophila melanogaster	48	200

Table 1: Simulated RNA-seq data-sets

net http://flux.sammeth.net/simulator.html

Of course, the simulated RNA-seq reads cover neither the whole sampled genome, nor all genes. Only "expressed" genes will have reads associated with their transcripts. Hence, some genomic mutations produced by the genome simulator will not be covered by any reads in fine. When describing the datasets, we indicate how many events of one categories has been seen by some reads. Intermediate scripts compute the positions and nature of all events (error, SNV, indels, normal and chimeric splice junctions) in the genome and all reads, so as to enable a precise evaluation of all predictions.

3 Simulated RNA-seq datasets

Here, we describe the simulated RNA-seq datasets used for comparing various tools (Table 1), and provide the numbers of alterations generated in each simulated genome, as well as those that are visible in the final RNA-seq reads (Table 2 and 3)

Type	SNV	Insertion	Deletion	Chimera
Genome-wide	3,139,937	287,336	287,502	1,002
Sequenced (75bp)	29,084	2,687	2,734	647
Sequenced (200bp)	52,971	4,810	4,901	914

Table 2: Number of mutations randomly generated in the simulated Human genome, and the numbers among those that were effectively sequenced in the Human simulated RNA-seq datasets from Table 1.

4 Tools used for comparison: version and parameters

We compared CRAC with other tools on its ability to map reads, to identify splice sites, to identify chimeric RNAs. The table 4 lists the software we used, their version, and parameters.

Type	SNV	Insertion	Deletion	Chimera
Genome-wide	132,193	12,146	12,168	676
Sequenced (75bp)	28,397	2,512	2,644	651
Sequenced (200bp)	30,549	2,698	2,861	668

Table 3: Number of mutations randomly generated in the simulated drosophila genome, and numbers of those that were effectively sequenced in the Drosophila simulated RNA-seq datasets from Table 1.

Figure 2: Distribution of $\log_2$(RPKM) for simulated Human data sets: *simulatedHuman75nt-42M* (in red) and *simulatedHuman200nt-48M* (in blue).

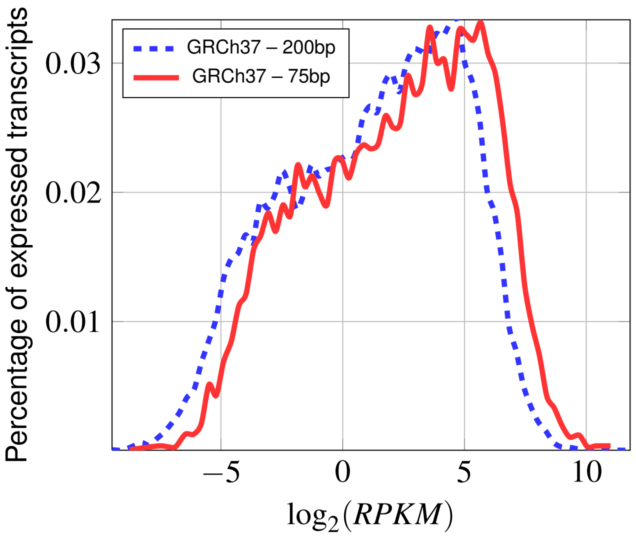

5 Score analysis to discriminate between sequence error and biological events

When CRAC analyzes a read, it needs to distinguish sequence errors from biological events using the support profile. Here, we detail the method we employed to derive a discrimination function that performs this distinction in the program. For this sake, we used a machine learning technique, called Support Vector Machines (SVM), which belongs to supervised classification approaches. Two SVMs were trained using simulated data for distinguishing: 1/ errors from mutations, 2/ errors from splice junctions. We explained how this was done in the first case; the same method has been used for the second.

Let us denote by f the discrimination function we want to learn. To do this, we use i/ the *support profile* (described in Algorithm section of the MS - section) to define two variables

Tool	Version	Parameters
Bowtie	0.12.7	–end-to-end –very-sensitive -k 2
Bowtie2	2.0.2	–best -n 3 -y -t -k 2
BWA	0.5.9	
BWA-SW	0.5.9	-b 5 -q2 -r1 -z10
CRAC	1.0.0	-k 22 -m 75
GASSST (short)	1.27	-w 18 -p 94 -s 3 -g 4
GASSST (long)	1.27	-w 18 -p 89 -s 3 -g 9
SOAP2	2.20	
GSNAP	2011-03-28	-N 1 –novel-doublesplices
MapSplice	1.15	–fusion -L 22
TopHat	1.2.0	
TopHat2	2.0.6	–b2-very-sensitive –fusion-search
TopHat-fusion	0.1.0 (BETA)	

Table 4: Parameters used for launching the tools. For CRAC, the `-m` parameter corresponds to the read length and is set according to the read set.

S_{in} and S_{out} of f, ii/ the *simulated dataset* (described Supplementary Section 2) to separate two labelled classes: "errors" and "biological events", iii/ a technique of supervised classification to compute f.

Support profile in a break As explained in the article, CRAC proceeds each read in turn and computes its k-mers *location profile* and k-mer *support profile*. Both a sequence error or a biological mutation (eg, substitution or indel) constitute a difference in sequence between the read and the genome, and consequently, both generate a *break* in the location profile. (see Figure 1a in the MS). To determine whether the source of this event is biological or erroneous, we must focus on the *support profile* (see Figure 1b). We compute two values: the average of the k-mers support outside the break, denoted by S_{out}, and the average of the k-mers support inside the break, denoted by S_{in}. The goal is to compare S_{in} and S_{out} with the following hypothesis: most reads that cover a biological event share the mutation, whereas an error occurs in a small number of reads.

Two labelled classes: "errors" and "biological events" On one hand, from the simulation protocol (supplementary section 2), we know which reads are affected by an error and which reads are affected by a biological event. On the other hand, using CRAC on simulated dataset (supplementary section 3), we can extract reads affected by an event by searching all breaks. As we know the answer for each event, we can define two labelled classes "errors" or "biological events" and save all pairs (S_{out}, S_{in}) for each class (one corresponding to sequence errors coordinates and the other corresponding to biological events coordinates).

Design of the separation To discriminate between the two classes, we use a technique of supervised classification called *Support Vector Machines* (SVM) [6]. It is a learning procedure that tries to find a discrimination function; this function can then be used to assign a new observation to the labelled classes. Here, we want to separate all pairs (S_{out}, S_{in}) for the two labelled classes "errors" and "biological events" according to a function f defined by $f(S_{out}) = S_{in}$. We used a SVM implementation in the R language (the package can be found at http://www.duclert.org/Aide-memoire-R/Apprentissage/SVM.php) with parameters set to:

`mode=CLASSIFY, kernel=POLYNOMIAL, degree=1/3`

to separate the two different vectors. Note that we used a polynomial kernel because the predicted separation was found to be a curve. We have only designed the function on the dataset hs-75 and we used the same for all dataset. In hs-75, we used an error model issued from an analysis of the Illumina® sequencing technology with real 75 nt reads [4] (see supplementary section 2.2). The function f computed by SVM to discriminate between sequence error and biological events (substitution or short indel) is the following:

$$f(S_{out}) = -2.40850 + 2.15859 \times S_{out}^{\frac{1}{3}} + 0.15670 \times S_{out}^{\frac{5}{6}} \tag{1}$$

Because a sequence error affects only a few nucleotides in a read, it can be easily distinguished from a splicing event (junction exon/intron) also characterized by a break. The predicted separation between sequence errors and splice events was found to be a different, less stringent curve. Thus, we decided to learn another specific SVM for splice junctions events with parameters set to (mode=CLASSIFY, kernel=POLYNOMIAL, degree=1/2). The function g computed by SVM to discriminate between sequence error and splicing events is:

$$g(S_{out}) = 0.51081 + 0.16758 \times S_{out}^{\frac{1}{2}} \tag{2}$$

Classification We can define several statistics:

TP: the true positives, *i.e.* the proportion of biological events which are classified correctly

FP: the false positives, *i.e.* the proportion of biological events which are misclassified

TN: the true negatives, *i.e.* the proportion of sequence errors events which are classified correctly

FN: the false negatives, *i.e.* the proportion of sequence errors events which are misclassified

Figure 3 illustrates the distribution of all points (S_{out}, S_{in}) for the two simulated Human datasets hs-75 and hs-200 (described in section 3). The black curves in Figure 3 (A) and Figure 3 (B) show the separations between errors and biological events calculated with SVM. Note that in case of sequence errors, we have a drop in the support profile (section) so it is natural that sequence errors are found under the curve. We visualize the formation of two separate clouds on each side of the curve (in both figures 3): TP (blue dots above the curve) and TN (blue dots below

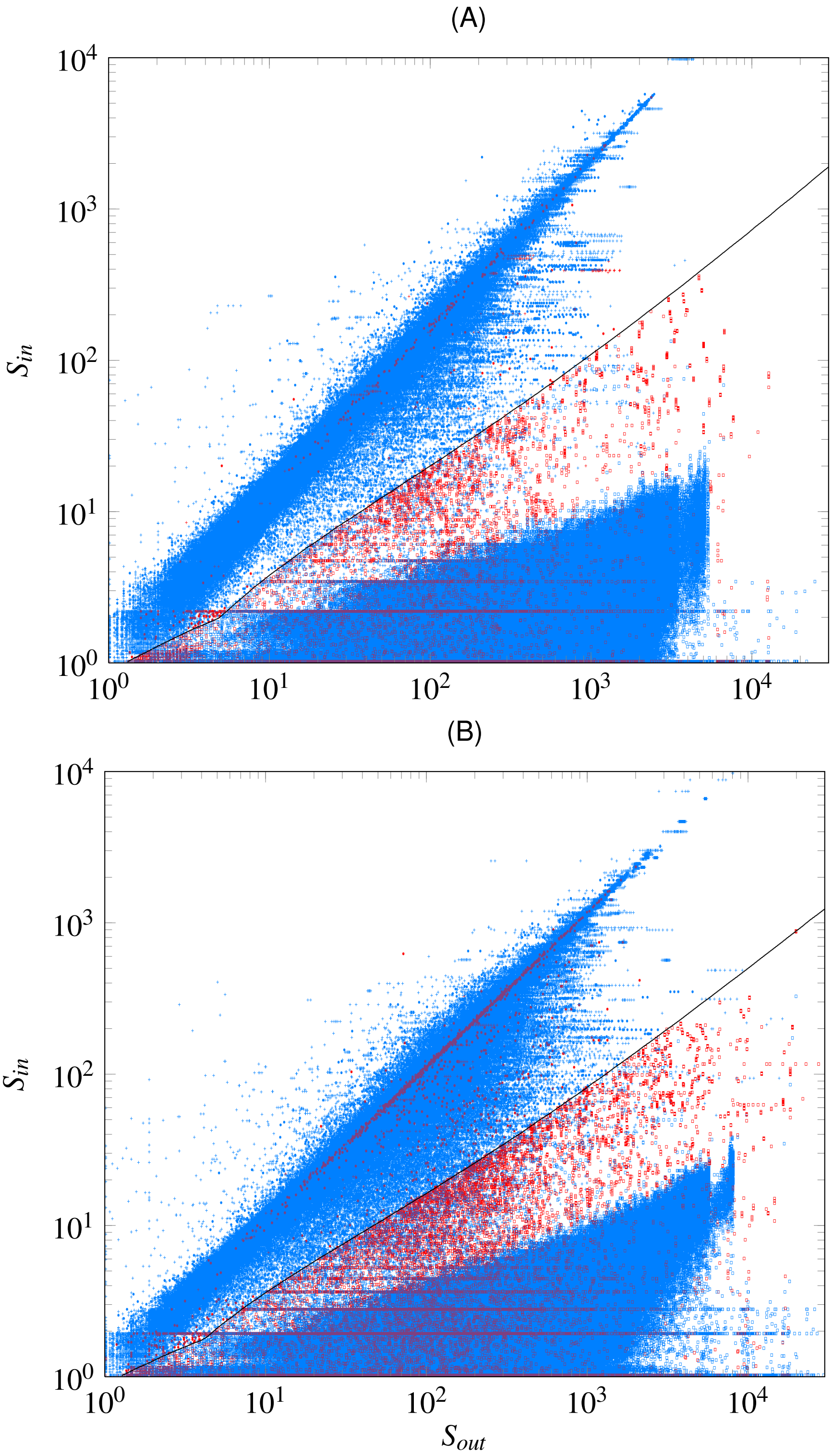

Figure 3: Computation of the separation between sequence errors and biological events for two read datasets: hs-75 (A) et hs-200 (B). Each point illustrates an event which is define in CRAC algorithm by a break. S_{out} is the average of the k-mers support outside the break and S_{in} is the the average of the k-mers support inside the break. The black curve is the separation, and was computed using a SVM approach. The red dots are misclassified points: FP are below the separation and the FN are above the separation. The blue points are well classified points: TP are above the separation and TN are below the separation.

the curve). On the contrary, the red dots represent the events that are misclassified: respectively FP for biological events and the FN for sequence errors. We can observe between the two clouds a mixture of blue dots and red dots that form an ambiguous zone where errors and biological events are indistinguishable.

However, our goal is to increase the precision in "biological events" class, *i.e.* to avoid sequence errors to be detected as biological events. In order to minimize the number of false positives in biological events, we used in the SVM model a variable precision parameter to increase precision for "biological events" class. We set that parameter with a probability of 0.98 even if in return it increases the number of FN for the class "errors".

Score in CRAC We establish a score for each event and for each break, using (S_{in}, S_{out}) . We compute $f(S_{out})$ and the vertical distance relatively to the separation $d = S_{in} - f(S_{out})$. Accordingly, we consider a sequence error when $d \leq 0$ and a biological event when $d > 0$. Indeed, a point which is close to the separation is less likely to be valid than a remote point. For example, a point with a $S1_{out} = 2.6$ and $S1_{in} = 1.5$ has a low score of 0.59 ($S1_{in} - f(S1_{out}) = 1.5 - (-2.40850 + 2.15859 \times 2.6^{\frac{1}{3}} + 0.15670 \times 2.6^{\frac{5}{6}}) = 1.5 - 0.91$). On the contrary, a point with a $S2_{out} = 26$ and $S2_{in} = 25$ will have a score of 23.85.

We see in Table 1 that CRAC has a better sensitivity in hs-200 while reads are longer (section), *i.e.* we find more errors and biological events. We can observe in Figure 3 more true positives in hs-200 than in hs-75 (blue dots above the curve). Because we have increased the variable precision parameter for the biological class, we can see in Figure 3 (A) and (B) that there are more FN than FP. In other words, TP, TN and FP are still the same between hs-75 and hs-200 (blue dots of each side and red dots above the curves) but not FN (red dots below the curve (B)). The results in Table 1 validate our approach because the precision of CRAC remains the same for hs-75 and hs-200.

In conclusion, we defined two functions: i/ one to distinguish sequence errors from point mutations (substitutions, short indels); ii/ another to discriminate splicing events in gray zone from the non-ambiguous zone. We have explained before how we design the function for i/ but the approach is the same for ii/.

6 Partition of chimeric RNAs

CRAC predicts candidate chimeric RNAs. These are splice junctions in which the 5' and 3' "exons" are either 1/ not located one after the other on the same chromosomal strand, these are said to be non colinear, or 2/ are too far apart on the chromosome to belong to the same gene. Obviously, the second case can only be determined by looking at annotations, which we forbid in CRAC. Without annotation, the decision between a splice inside one gene or across two genes is made arbitrarily depending on the distance on the chromosome. For simplicity, we use the term "exon" although the transcribed region may be located outside a known gene, in an intron, in antisense of a gene. By exon, we mean an unspliced part of the RNA.

CRAC further partitions all chRNA in five classes depending on the exon organization; this partition resembles that depicted in [7, Figure 1]. The five classes are as follows:

1. The exons are located on different chromosomes
2. The exons are colinear but (likely) belong to different genes; this must be checked with annotation.
3. The exons are on the same chromosome and same strand, but not in the order in which they are found on DNA, and they do not overlap each other.
4. The exons are on the same chromosome but on different strands.
5. Exactly as in class 3, but the exons overlap each other by at least one nucleotide.

In class 1, the splicing joins pieces from distinct chromosomes, while in classes $2-5$ the exons are on the same chromosome. In summary, class 2 is the only colinear case.

We create class 5 to distinguish cases truely due to large scale inversions (class 3) from those likely due to local inversions or repeats inside genes. When analyzing the breast cancer libraries, we found many such cases.

To investigate more closely these candidates, we confront them to Ensembl annotations [3] and could determine whether the involved "exons" are in annotated exons, introns, or outside genes.

References

[1] Philippe, N., Boureux, A., Tarhio, J., Bréhélin, L., Commes, T., and Rivals, E. Using reads to annotate the genome: influence of length, background distribution, and sequence errors on prediction capacity. *Nucleic Acids Res.* **37**(15), e104 (2009). 2

[2] Griebel, T., Zacher, B., Ribeca, P., Raineri, E., Lacroix, V., Guigó, R., and Sammeth, M. Modelling and simulating generic rna-seq experiments with the flux simulator. *Nucleic Acids Res.* **40**(20), 10073–10083 (2012). 3

[3] Flicek, P., Aken, B. L., Beal, K., Ballester, B., Caccamo, M., Chen, Y., Clarke, L., Coates, G., Cunningham, F., Cutts, T., Down, T., Dyer, S. C., Eyre, T., Fitzgerald, S., Fernandez-Banet, J., Graf, S., Haider, S., Hammond, M., Holland, R., Howe, K. L., Howe, K., Johnson, N., Jenkinson, A., Kahari, A., Keefe, D., Kokocinski, F., Kulesha, E., Lawson, D., Longden, I., Megy, K., Meidl, P., Overduin, B., Parker, A., Pritchard, B., Prlic, A., Rice, S., Rios, D., Schuster, M., Sealy, I., Slater, G., Smedley, D., Spudich, G., Trevanion, S., Vilella, A. J., Vogel, J., White, S., Wood, M., Birney, E., Cox, T., Curwen, V., Durbin, R., Fernandez-Suarez, X. M., Herrero, J., Hubbard, T. J. P., Kasprzyk, A., Proctor, G., Smith, J., Ureta-Vidal, A., and Searle, S. Ensembl 2008. *Nucleic Acids Res.* **36**(S1), D707–714 (2008). 4, 11

[4] Aury, J., Cruaud, C., Barbe, V., Rogier, O., Mangenot, S., Samson, G., Poulain, J., Anthouard, V., Scarpelli, C., Artiguenave, F., and Wincker, P. High quality draft sequences for prokaryotic genomes using a mix of new sequencing technologies. *BMC Genomics* **9**, 603 (2008). PMID: 19087275. 4, 8

[5] Mortazavi, A., Williams, B. A., McCue, K., Schaeffer, L., and Wold, B. Mapping and quantifying mammalian transcriptomes by RNA-Seq. *Nat. Methods* **5**(7), 621–628 (2008). 4

[6] Crammer, K. and Singer, Y. On the algorithmic implementation of multiclass kernel-based vector machines. *Journal of Machine Learning Research* **2**, 265–292, December (2001). 8

[7] Gingeras, T. Implications of chimaeric non-co-linear transcripts. *Nature* **461**, 206–11 (2009). 11
