# Supplementary material for: CRAC: an integrated approach to the analysis of RNA-seq reads
Source: Genome Biol. 2013 Mar 28;14(3):R30. doi: 10.1186/gb-2013-14-3-r30 (PMC4053775; doi:10.1186/gb-2013-14-3-r30)
Supplement: Additional file 3 — Results for simulated RNA-seq data. [file gb-2013-14-3-r30-S3.PDF]

CRAC: An integrated approach to analyse RNA-seq reads
Additional File 3
Results on simulated RNA-seq data.

Nicolas Philippe and Mikael Salson and Thérèse Commes and Eric Rivals

February 13, 2013

1 Results on Drosophila simulated RNA-seq data

All analyses were performed on sets of Human and Drosophila simulated RNA-seq data to assess the impact of the reference genome. Results on Human data are presented in the manuscript, while all pendant results on Drosophila datasets are given here. Although Drosophila and Human genomes differ in length, gene density as well as in number of short introns, the results are similar between the two species for mapping, splice junction or chimeric RNA predictions.

Mapping Figure 1 compares the sensitivity and precision of mapping between Bowtie, BWA / BWA-SW, CRAC, GASSST, GSNAP and SOAP2 [1, 2, 3, 4, 5]. The version and parameters used for these programs are given in Additional File 2. As for Human data, the percentages of uncorrectly mapped reads (in red) are almost invisible except for BWA-SW on 200 nt reads, meaning that almost all output genomic locations are correct. However, the difference in sensitivity remains and shows that CRAC exhibits both high sensitivity and precision. Again, its behavior improves with longer reads.

Figure 1: Comparison of **sensitivity** and **precision** on simulated RNA-seq against the drosophila genome for (A) *simulatedDroso75nt-45M* and (B) *simulatedDroso200nt-48M*.

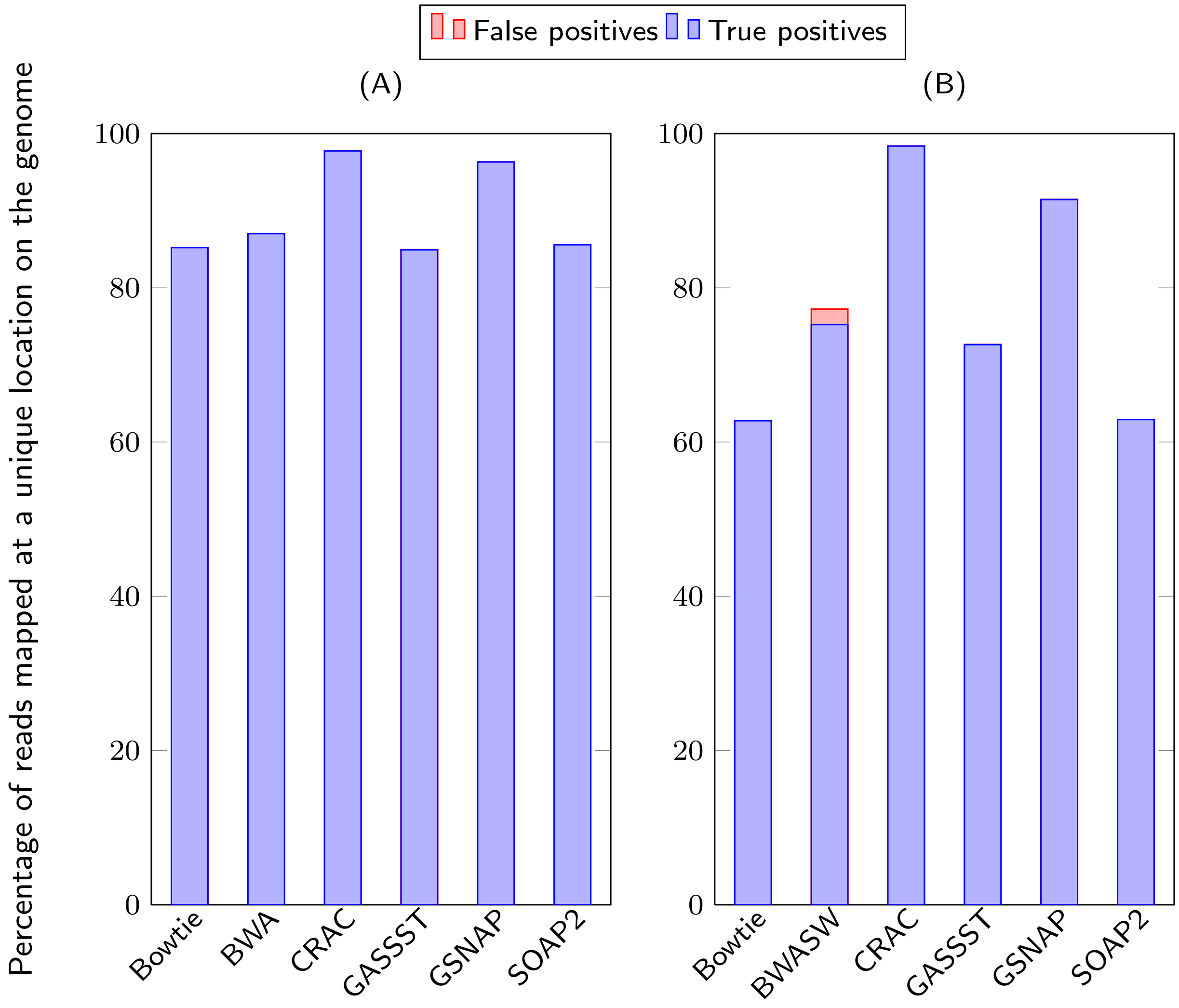

Normal and chimeric splice junctions detection. Table 1 shows the sensitivity and precision of splice junction prediction on *D. melanogaster* simulated data. CRAC is compared to TopHat, MapSplice, and GSNAP [6, 7, 8]. Again CRAC is highly sensitive, even if TopHat achieves between +2 to +4 points in sensitivity, but CRAC remains the most precise among all tools. For instance, TopHat yields 10 to 20 times more false positive junctions than CRAC.

Table 1: Sensitivity and precision of detection of splices among different softwares. TP is the number of true positives and FP the number of false positives.

Tool	75bp				200bp			
	Sensitivity	Precision	TP	FP	Sensitivity	Precision	TP	FP
CRAC	87.31	**99.78**	39,637	84	91.15	**99.59**	42,835	178
GSNAP	80.67	99.05	36,623	350	79.7	98.8	37,453	454
MapSplice	86.19	99.54	39,127	182	89.31	99.42	41,971	244
TopHat	**91.04**	95.94	41,329	1,749	**93.89**	94.93	44,123	2,354

Table 2 shows the sensitivity and precision of chimeric junction prediction on *D. melanogaster* simulated data. CRAC is compared to MapSplice [7], TopHat-fusion [9], and TopHat-fusion-Post (*i.e.*, TopHat-fusion followed by a post-processing script).

Here, both CRAC and TopHat-fusion achieve better sensitivity than on Human data. However, CRAC reaches much higher precision than any other tool, at the exception of TopHat-fusion-Post which has 100% precision but delivers only 2 candidate chimeric junctions, that is $< 1\%$ sensitivity.

Table 2: Sensitivity and precision of detection of chimera among different softwares. TP is the number of true positives and FP the number of false positives.

Tool	75bp				200bp			
	Sensitivity	Precision	TP	FP	Sensitivity	Precision	TP	FP
CRAC	75.94	99.8	1,069	2	**68.29**	**99.1**	1,217	11
MapSplice	3.63	36.45	51	89	3.2	0.19	57	29,784
TopHatFusion	**82.35**	47.13	1,157	1,298				
TopHatFusionPost	0.14	**100**	2	0				

2 Additional results on Human simulated RNA-seq data

2.1 Comparison of 11 vs 42 million reads

We assessed the impact on mapping results of the size of the dataset in terms of number of reads, and hence of coverage. We performed the same analysis with a subset of 11 million reads and with the whole set of 42 million reads. The read length is 75 nt. The results for each set and for all tools are displayed in Figure 2 (A) for 11 millions and (B) for 42 millions reads. The impact is negligible, except for BWA that yields more false locations (small red bar on top of the blue one in A) with the medium size set (96.28 vs 99.13%). Especially, CRAC sensitivity and precision are not impacted by the number of reads, although this number changes the support values. For comparison, as shown in the manuscript, using longer reads impacts much deeply all mapping tools (Figure 3 in the MS).

2.2 Comparison of running times and memory usages

We give in Table 3 the running times and memory usages observed for mapping and splice junction prediction with various programs for processing the 42 million of 75 nt reads (Human simulated data). Times can be in days (d), hours (h) or even minutes (m), while the amount of main memory is given in Gigabytes (Gb). Although CRAC performs several prediction tasks - for point mutations, indels, splice junction and chimeric RNAs - its running time is longer than those of mapping tools and shorter than those of splice junction prediction tools. Its memory consumption is larger due to the use of a read index, the Gk arrays. This index is indispensable to query the support profile of each read on the fly.

Programs	Bowtie	BWA	GASSST	SOAP2	CRAC	GSNAP	MapSplice	TopHat
Time (dhm)	7h	6h	5h	40m	9h	2d	4h	12h
Memory (Gb)	3	2	43	5	38	5	3	2

Table 3: Running times and memory usages observed for mapping or splice junction prediction with various programs.

3 Cases of failures

For some simulated datasets, we experienced failures while running other tools in our comparisons, as mentioned in the Results of the article. For instance, TopHat-fusion did not deliver results on the 200 nt read datasets [9]. TopHat-fusion was unable to process the 200 nt simulated reads for a yet unknown reason. On that input, TopHat-fusion ran during about one month, while still filling temporary files but it stopped without any error message. We tried a few times and always obtained the same results. Finally, we contacted TopHat-fusion's contributors twice *via* their mailing list, but did not obtain any reply.

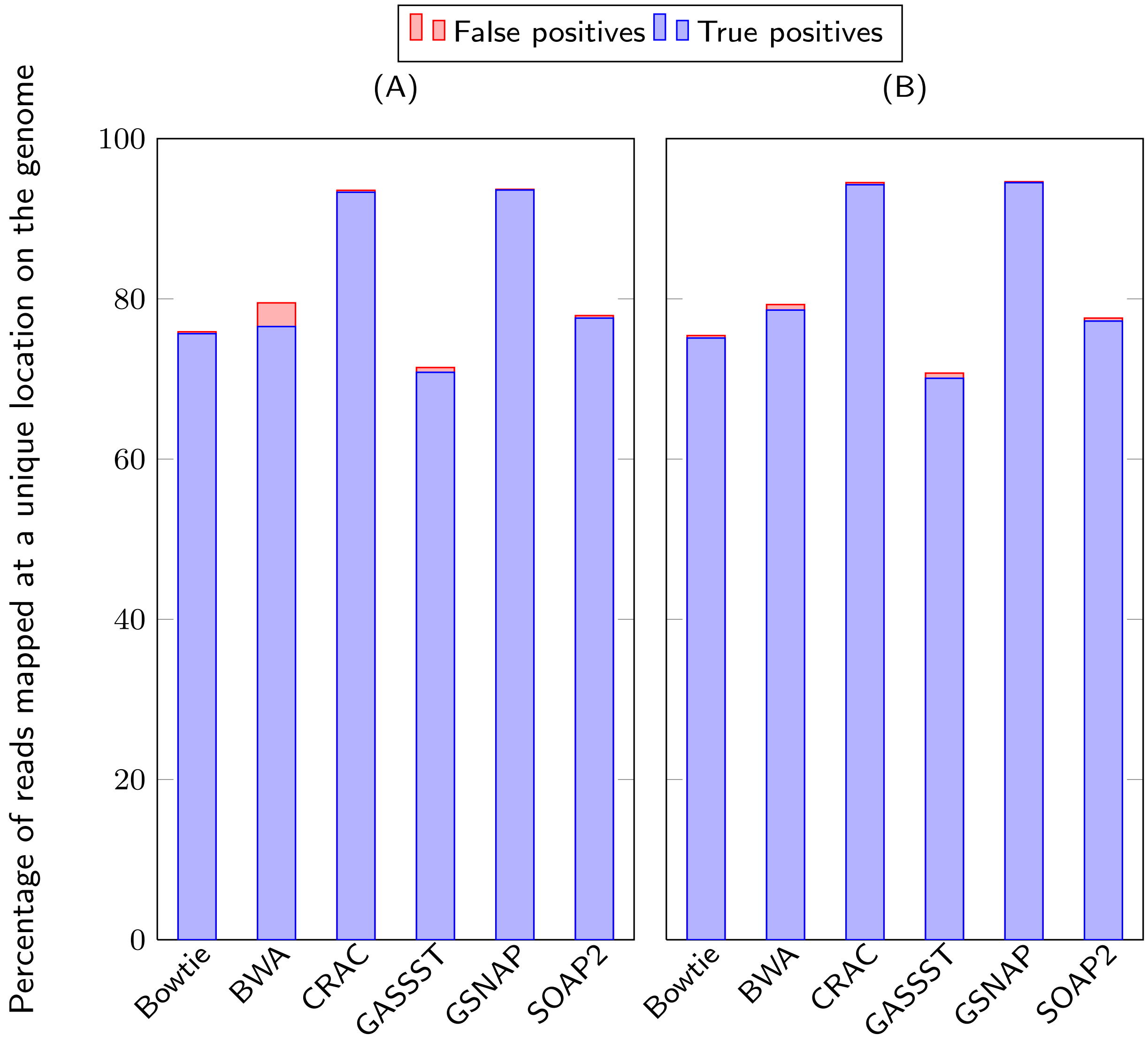

Figure 2: Impact on mapping results of medium (A) versus large (B) dataset. Comparison of **sensitivity** and **precision** on simulated RNA-seq against the Human genome on medium and large size datasets (11M-75 nt vs 42M-75 nt).

References

[1] Li, H. and Durbin, R. Fast and accurate short read alignment with Burrows-Wheeler transform. *Bioinformatics* **25**(14), 1754–1760 (2009). 1

[2] Li, H. and Durbin, R. Fast and accurate long-read alignment with Burrows-Wheeler transform. *Bioinformatics* **26**(5), 589–595 (2010). 1

[3] Li, R., Li, Y., Kristiansen, K., and Wang, J. SOAP: short oligonucleotide alignment program.

Bioinformatics **24**(5), 713–714 (2008). 1

[4] Langmead, B., Trapnell, C., Pop, M., and Salzberg, S. Ultrafast and memory-efficient alignment of short DNA sequences to the human genome. *Genome Biol.* **10**(3), R25 (2009). 1

[5] Rizk, G. and Lavenier, D. GASSST: global alignment short sequence search tool. *Bioinformatics* **26**(20), 2534 –2540 (2010). 1

[6] Trapnell, C., Pachter, L., and Steven L. Salzberg. TopHat: discovering splice junctions with RNA-Seq. *Bioinformatics* **25**(9), 1105–1111 (2009). 3

[7] Wang, K., Singh, D., Zeng, Z., Coleman, S. J., Huang, Y., Savich, G. L., He, X., Mieczkowski, P., Grimm, S. A., Perou, C. M., MacLeod, J. N., Chiang, D. Y., Prins, J. F., and Liu, J. MapSplice: accurate mapping of RNA-seq reads for splice junction discovery. *Nucleic Acids Res.* **38**(18), e178 (2010). 3

[8] Wu, T. D. and Nacu, S. Fast and SNP-tolerant detection of complex variants and splicing in short reads. *Bioinformatics* **26**(7), 873 –881 (2010). 3

[9] Kim, D. and Salzberg, S. L. TopHat-Fusion: an algorithm for discovery of novel fusion transcripts. *Genome Biol.* **12**(8), R72 (2011). 3, 4
