# Supplementary material for: CRAC: an integrated approach to the analysis of RNA-seq reads
Source: Genome Biol. 2013 Mar 28;14(3):R30. doi: 10.1186/gb-2013-14-3-r30 (PMC4053775; doi:10.1186/gb-2013-14-3-r30)
Supplement: Additional file 4 — Results for real RNA-seq data. [file gb-2013-14-3-r30-S4.PDF]

CRAC: An integrated approach to analyse RNA-seq reads
Additional File 4
Results on real RNA-seq data.

Nicolas Philippe and Mikael Salson and Thérèse Commes and Eric Rivals

February 13, 2013

1 The real RNA-seq data sets

Five distinct Human RNA-seq datasets were used for assessing the capacity of predicting splice junctions and chimeric RNAs from CRAC and other tools. The main characteristics of these data sets are summarized in Table 1. The first four lines are breast cancer libraries sequenced using unstranded paired-end RNA-seq from Edgren *et al.* [1]. The last line, ERR030856, corresponds to a normal multi-tissue library sequenced using stranded RNA-seq.

Data source	Library	Read type	Fragment length	Read length	Number of fragments (or reads)
Breast cancer libraries [1]	BT474	Paired	100-200	50	21,423,697
	SKBR3	Paired	100-200	50	18,140,246
	KPL4	Paired	100	50	6,796,443
	MCF7	Paired	100	50	8,409,785
ERR030856	16 normal tissue mixtures	Single	-	100	75,000,000

Table 1: Real Human RNA-seq data used to compare splice and chimeras detection tools: four breast cancer libraries of [1] of unoriented 50 nt reads, sequenced with 1G Illumina Genome Analyzer 2X, and accessible at NCBI Sequence Read Archive [SRA:SRP003186]; one collection of 100 nt oriented reads sequenced with HiSeq 2000 Illumina® from 16 normal tissues mixtures from 11 adult individuals of widespread ages ([19; 86]) from Experiment E-MTAB-513 of Illumina bodyMap2 transcriptome (see details at http://www.ebi.ac.uk/arrayexpress/experiments/E-MTAB-513; this collection is accessible at http://trace.ddbj.nig.ac.jp/DRASearch/experiment?acc=ERX011226.

The tools, versions and parameters used for the comparison in all analyses are given in Table 4 of Additional File 2.

2 Predicting splice junctions on real RNA-seq data

Four programs, CRAC, TopHat, GSNAP, and MapSplice were launched to predict splice junctions on a data set of 75 million stranded 100 nt reads (ERR30856). Splice junctions were then confronted to Human RefSeq transcripts to determine whether positions found coincide with start/end of known RefSeq exons. Found junctions were partitioned into *known*, *new* and *other* junctions (see the main manuscript for a definition). We determined the intersections between the set of predicted junctions for any combination of tools. The agreement, *i.e.* the size of these intersections, are displayed in the form of Venn diagrams. These plots were obtained using Venny at `http://bioinfogp.cnb.csic.es/tools/venny/index.html`.

Figures 1 and 2 show the agreement between the predictions of each tool respectively on novel junctions, and on multi-exon RefSeq transcript for which at least one known or novel splice junction was detected.

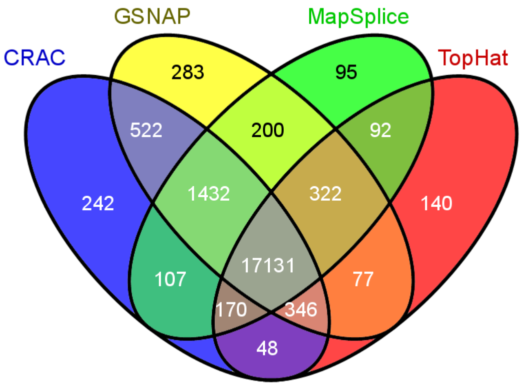

Figure 1: Venn diagram showing the agreement among tools on known junctions using known RefSeq transcripts on the ERR030856 Human dataset.

2.1 Identifying reads covering small exons

Thanks to its *k*-mer profiling approach, CRAC can detect reads that covers multiple adjacent splice junctions in the same transcript, and therefore includes entirely some small exons. CRAC identifies several breaks in the location profile of such reads and determines the donor and acceptor genomic positions of each junction. An example of read that covers two adjacent junctions

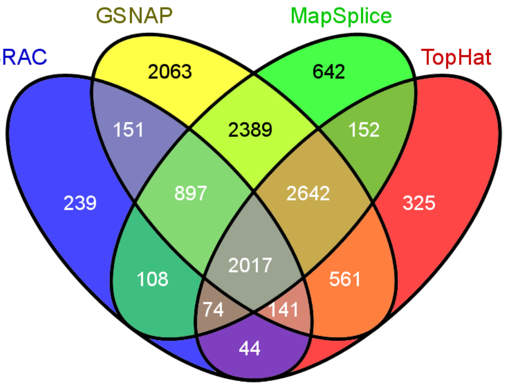

Figure 2: Venn diagram showing the agreement among tools on new splice junctions using known RefSeq exons on the ERR030856 Human dataset.

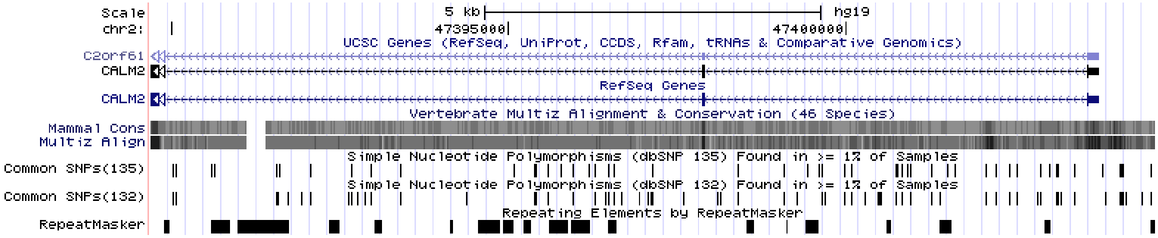

Figure 3: A read spanning three exons and two splice junctions of human Calmodulin 2 (CALM2) gene. This graphical view was obtained from the UCSC genome browser.

and incorporates a 29 nt exon of the Human calmodulin 2 gene (CALM2) is illustrated in Figure3 as viewed in the UCSC genome browser at `http://genome.ucsc.edu/cgi-bin/hgTracks?org=human`.

2.2 Agreement on splice junctions found by CRAC, TopHat, GSNAP, and MapSplice on the ERR030856 library

We predicted splice junctions on the ERR030856 library with each of CRAC, GSNAP, MapSplice, and TopHat (see Results in the main manuscript). First, we investigated the agreement between these four tools on *Known Junctions* (KJ) in the tables 3 and 2. Table 3 gives the number of junctions reported by each tool, as well as percentages of junctions in the intersection of all four tools, or among the three tools that perform best on this dataset (CRAC, GSNAP, MapSplice). As commented in the manuscript, we observed a large agreement among them. For more details, we also computed the numbers and percentages of KJ that are specific to each tool,

	CRAC	GSNAP	MapSplice	TopHat
Total	142,000	144,180	140,876	116,687
Shared %	97.41	97.53	99.42	98.83
Shared C-G-M %	89	87	89	NA
Shared all %	72	70	72	87

Table 2: Agreement on Known Junctions (KJ) predicted on library ERR030856 by four tools. *Total*: number of reported KJ. *Shared %*: percentage of KJ shared with at least one other tool. *Shared C-G-M*: percentage of KJ shared by CRAC, GSNAP, MapSplice. NA: not applicable. *Shared all*: percentage of KJ shared with all three other tools. For each tool, $> 97\%$ of the KJ it finds are also predicted by one other program. The agreement on well annotated junctions is larger among CRAC, GSNAP, MapSplice, than with TopHat; this is explained by the fact that TopHat finds $\simeq 25,000$ splice junctions less than the other tools.

or in the intersection of any combination of tools; see Table 2.

Finally, we computed the percentage of known junctions found by CRAC that are also reported by the other tools. We then focused on i/ reads covering entirely small exons and ii/ KJ with a large intron reported by CRAC. We computed for each category, how many items the other tools were able to report. Results are displayed in Table 4, where we also calculated the probability that a given tool found that many reads/junctions or less. The probability is computed assuming a binomial distribution and therefore assuming that the category considered represents a random sample of known junctions.

2.3 Further investigations on junctions

If the four tools show a good agreement on known junctions, it is less the case with new junctions and other junctions. Regarding other junctions, we cannot rely on RefSeq annotations to infer canonical junctions that would easily be comparable among the four tools.

To circumvent those problems, we performed another experiment that should give more insights on the predictions made by the four tools. We used the predictions made by the four tools to extract a genomic sequence of 25 nt upstream and 25 nt downstream of the junction. The 50 nt sequence is then Blasted against both the human mRNA refseq [2] and the human ESTs [3]. Blastn was launched using the following options `-F F -W 15 -a3 -G 5 -E 3 -e 0.001 -w -1 -B 1`. For obvious reasons, there are much more hits on the ESTs than on mRNA RefSeq. Therefore in the following we only report hits on ESTs. Good hits, with low E-values ($\leq 10^{-15}$), witness the fact that a predicted junction is found with high confidence, (almost) exactly on existing ESTs. Good hits should be taken as additional evidence rather than as a guarantee of the existence of this junction. On the other hand, in hits with high E-values ($\geq 10^{-10}$), only one half of

[2] Recovered using `homo sapiens[organism] AND mrna [Filter] AND refseq [Filter]` on http://www.ncbi.nlm.nih.gov/nuccore.

[3] Recovered from http://www.ncbi.nlm.nih.gov/nucest/?term=homosapiens[organism] and filtered out identical sequences resulting in 8,469,118 distinct sequences.

	Known Junctions only found by				Intersection of the junctions found by										
	CRAC	GSNAP	MapSplice	TopHat	C-G	C-M	C-T	G-M	G-T	M-T	C-G-M	C-G-T	C-M-T	G-M-T	All
KJ #	3,683	3,565	815	1,370	2,500	2,019	775	2,418	951	852	25,170	3,137	3,163	4,886	101,553
CRAC %	2.59				1.76	1.42	0.55				17.73	2.21	2.23		71.52
GSNAP %		2.47			1.73			1.68	0.66		17.46	2.18		3.39	70.43
MapSplice %			0.58			1.43		1.72		0.6	17.87		2.25	3.47	72.09
TopHat %				1.17			0.66		0.82	0.73		2.69	2.71	4.19	87.03

Table 3: Agreement on Known Junctions (KJ) predicted on library ERR030856 by four tools: detailed figures for any combination of tools. *KJ #*: number of KJ found specifically by a tool or a combination of tools. *Tool %*: percentage of the corresponding combination (in column) over the total found by the tool on that line. Empty columns are combination not including that tool. A combination of tools is denoted by the initials of the corresponding programs: for instance, the combination C-G-T corresponds to junctions found by CRAC, GSNAP, and TopHat.

	CRAC	GSNAP	MapSplice	TopHat
Agreement with CRAC %	100	93	93	76
Reads covering two KJ	9,817	8,338	9,167	7,496
Probability		9.61×10^{-178}	0.972	0.374
Reads covering three KJ	89	34	78	52
Probability		2.36×10^{-41}	5.09×10^{-2}	1.20×10^{-4}
KJ with intron $\geq$ 100 Knt	752	695	589 [1]	470
Probability		0.212	2.06×10^{-3}	6.46×10^{-18}

Table 4: Finding read covering multiple Known splice Junctions (KJ) and KJ with large introns. Ratio of KJ found by CRAC and also reported by the other tool. In the prediction of CRAC, we consider first the reads that cover two or three KJ (such reads include entirely one or more exons), and then KJ with large introns. Among the reads, respectively KJ, found by CRAC, we computed how much are also reported by the tool in that column, as well as the probability that it finds that many reads or less, according to its global agreement with CRAC. The probability says if the tool does at least as good at finding such reads/junctions as one would expect given its agreement with CRAC. For most of the category, GSNAP, MapSplice, and TopHat find less reads/junctions than CRAC. However, *e.g.* MapSplice and TopHat find about as much reads covering 2 exons as expected “by chance” ($p > 0.05$), while GSNAP finds significantly less than expected. All tools find less than expected reads covering three junctions, while MapSplice, and TopHat find less KJ with large introns than expected.

MapSplice, due to the default parameters, was not able to report junctions with an intron $\geq$ 200 knt. In the probability calculation we therefore removed 96 junctions reported by CRAC, that have such a large intron.

	CRAC	GSNAP	MapSlice	TopHat	All but CRAC
Aligned	115	704	258	131	1 056
Percentage aligned	48 %	34 %	40 %	40 %	40 %

Table 5: Absolute and relative numbers of new junctions only predicted by CRAC, GSNAP, MapSplice or TopHat that were aligned to human ESTs with an E-value $\leq 10^{-15}$ or junctions that where predicted by all tools but CRAC.

	CRAC	GSNAP	MapSlice	TopHat
Aligned	11 395	15 975	13 907	11 579
Percentage aligned	69 %	47 %	50 %	44 %

Table 6: Absolute and relative numbers of other junctions predicted by CRAC, GSNAP, MapSplice or TopHat that were aligned to human ESTs with an E-value $\leq 10^{-15}$.

the junction has been aligned. Such hits demonstrate that the predicted junction was not seen in the whole collection of human ESTs, and are therefore likely to be false positives.

2.3.1 Blasting specific new junctions

Since there exists a discrepancy among the predictions of new junctions, we started by blasting them. More specifically, we focus on junctions that are detected by only one tool. Since the intersection between GSNAP, MapSplice and TopHat is the largest one, we also take into account junctions from that set.

CRAC yields less new junctions that are specific to it compared to GSNAP or MapSplice, but, as can be seen in Table 5, CRAC is more accurate than concurrent methods. Predictions made by the other tools are slightly less reliable than CRAC's. On the other hand, CRAC delivers less predicted junctions of that specific category than the other tools. For reasons explaining that, see section 2.4.

2.3.2 Blasting other junctions

We also reproduced the experiment on the sets of other junctions of each tool. We also focus on high quality hits having an E-value lower than or equal to 10^{-15}. The results are presented in Table 6. We observe that GSNAP and MapSplice have the highest number of high quality alignments, while CRAC has the highest proportion.

2.3.3 Blasting all junctions

Since the separation between known, new and other junctions is somehow arbitrary, and is relative to RefSeq, it is also interesting to consider all junctions predicted by a tool altogether to assess each tool's performance. As a summary we made two plots, in Figure 4. We notice that GSNAP predicts more high quality hits (159,702), followed by MapSplice (152,957), followed

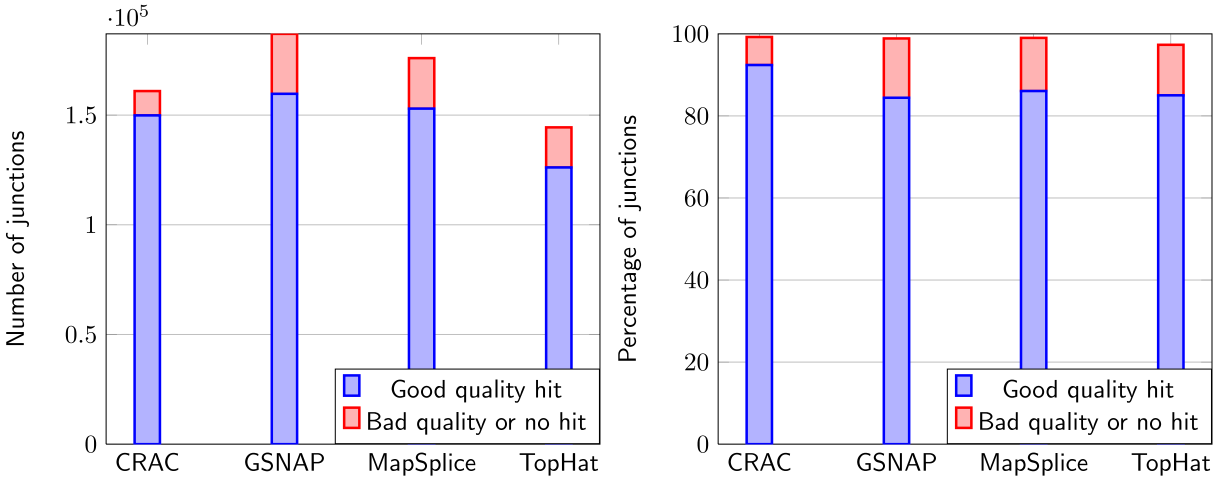

Figure 4: Aligning all the junctions from CRAC, GSNAP, MapSplice, TopHat to human ESTs using BlastN. Hits with E-values $\leq 10^{-15}$ are considered as good quality hits whereas hits with E-values $\geq 10^{-10}$ or junctions that were not aligned are considered as bad quality or no hit. Figures are provided first with absolute numbers (number of distinct junctions) and then as a percentage of the total number of distinct junctions.

by CRAC (149,872) and TopHat (126,143). CRAC is characterised by a low proportion of bad quality hits (6.8 %) versus 14 % for GSNAP, 13 % for MapSplice and 12 % for TopHat.

2.4 Investigating new junctions unmapped by CRAC

To understand why CRAC had its worst performances with the new junctions, we analyse a random sample drawn from the junctions predicted by the three other tools together. Twenty-one junctions are sampled out of 2,642, and the corresponding read where they appear are considered for a manual analysis. Of these junctions, nineteen are weakly expressed alternative transcripts. Meaning that these specific junctions are rare but the involved exons also participate in other junctions, that are much more expressed. Therefore CRAC identifies a variation in the support profile (the exons are well expressed, but the junction is poorly expressed) and considers that it may consist of a sequencing error. However CRAC is aware that this kind of error is unusual for a sequencing error. That is why CRAC classifies sixteen of these cases as an "undetermined error" and gives more clue by stating that it is probably a splicing event (the positions of the event are also given).

2.5 Testing junction prediction on negative controls.

We report in the Results section of the MS, the output of CRAC on a set of negative controls splice junctions obtained by associating true RefSeq exons. The command line used for running CRAC is:

```
crac -i GRCh37 -r random-refseq-junction-reads-100k.fa -k 22 -m 76
```

```
--splice random-refseq-junction-reads-100k-GRCh37-22.splice
--nb-threads 2 -n 15 --max-splice-length 300000 --max-duplication 5
 --min-percent-duplication-loc 0.5 --min-loc-repetition 2
```

The collection of reads used as negative controls is available at: `http://crac.gforge.inria.fr/`

3 Predicting chimeric RNAs on four breast cancer libraries.

3.1 Parameters for CRAC

To test CRAC on real data regarding the identification of chimeric RNA (chRNA), we compared its results to the findings of Edgren *et al.* [1] and of TopHat-fusion on four breast cancer RNA-seq libraries. These were published in Edgren [1] and also analysed in TopHat-fusion [2]. Contrarily to the other data we used, either simulated or real, these RNA-seq libraries contain shorter reads: 50 nt. Hence, we needed to adapt CRAC's parameters to take this shorther length into account. We alter two parameters:

- the number of adjacent k-mers that must consistently indicate the same unique location in a read was decreased from 15% to 10% of the read length, that is from 7 to 5 (`-min-percent-single-loc 0.10`)
- the number of k-mers adjacent to each side of the break border whose location is checked for concordance was lowered to 2 instead of 10 (`-max-extension-length 2`). This parameter is used during the break fusion procedure to determine whether we face a colinear (*i.e.*, normal) rather than a chimeric splice junction.

We used $k = 22$, as for the other analyses to avoid an increase in false locations; all other parameters were left by default or as for the other analyzes (see Table 4 of Additional File 2).

We used stringent criteria for predicting chRNA, which is done by setting the following parameters:

```
chimera_break >= k-1-(5)
min_support_in_chimera >= 2
max_extension_for_find_single_loc =5 for each border break
```

3.2 Filtering for normal splice junctions with GSNAP

We filtered the chRNA predicted by both CRAC and TopHat-fusion using GSNAP to avoid those that could have a continuous or colinear splice alignment with slightly less identities. Such an alignment represents an alternative to the detected chimeric alignment. Thus, we consider such candidates to be less robust. For this filtering, we set the parameters that enable GSNAP to detect splice junctions in individual reads, *i.e.* the `--novelsplicing (or -N)` flag. All other options were set to default.

3.3 Rerunning TopHat-fusion

In the article, we report several recurrent chRNAs detected by CRAC but not found by TopHat-fusion. We sought to understand the reasons of this difference, especially if TopHat-fusion detects these chimeric junctions based on alignment criteria, but then filter them out based on biological knowledge. As TopHat-fusion reports first the set of reads that generates the initial hits (in file `accepted_hits.sam`) before its internal filtration step, it is possible to answer this question. For this sake, we ran TopHat-fusion on the four libraries as described in their article [2], and searched all detected chRNAs in its intermediate file.

Parameters of TopHat-fusion: `--fusion-anchor-length 20`

3.4 Running times for the breast cancer libraries

Table 7 gives the running times of CRAC and TopHat-fusion to analyze each of the four breast cancer libraries of 50 nt reads. CRAC is between 5 and 10 times faster than TopHat-fusion.

Breast cancer libraries [1]	BT-474	KPL-4	MCF-7	SK-BR-3
CRAC	1h50m	41m	54m	1h05m
TopHat-fusion	11h58m	3h28m	4h22m	11h12m

Table 7: CPU time for CRAC and TopHat-fusion to process with 4 threads the Breast cancer libraries BT-474, KPL-4, MCF-7 and SK-BR-3 from [1].

3.5 Distribution of candidate chimeric RNA found by CRAC

CRAC predicted 455 candidate chRNAs that are partitioned in five classes, as explained in Section 6 of Additional File 2. Class 2 candidates represent only two percents of the total, thereby showing that, although arbitrary, the threshold used to distinguish between splice inside one gene or across distinct genes, works reasonably for Human data. Annotations show that some of these cases are indeed normal splice junctions inside a known gene.

Class	Nb	Total	Proportion
1	118	455	0.26
2	10	455	0.02
3	109	455	0.24
4	127	455	0.28
5	91	455	0.20

3.6 Case candidate "chimeric" RNA with internal repeat located inside LONP1 gene

This candidate chRNA is identified in class 5: it appears as an inversion because of an internal repeat. We use the term "chimeric" simply because such reads cannot be explained with sim-

ple colinear alignments. It means "non colinear" and makes no assumption about underlying mechanisms.

Figure 5 shows the analysis of one of the reads that gave rise to this prediction. Neither can it be mapped continuously on the genome, nor did GSNAP find a continuous alignment for it. Instead, it is mapped as a chimeric read with a small scale inversion on chromosome 19 minus strand in two parts depicted in blue and yellow. The k-mer location profile exhibited a break after the blue part, and the first located k-mer after the break is at the start of the yellow part. The blue part ends at position $5,692,012$, while the yellow part starts at position $5,691,992$, *i.e.* slightly before. Hence, CRAC classifies it as a chimera with inversion. Both parts overlap on the chromosome 19, which implies that the read contains a sequence repeated twice $TCA\ldots AGA$ (shown in boldface below). This chimeric alignment is confirmed by BLAT (below), which finds exactly the same junction point.

This duplication could be due to a known variant. We thus searched for possible known variants in this chromosomal region in eight distinct Human genomes on Ensembl, but find none [3]. However, we observed this chimeric junction, but also found the same junction without the duplication in other libraries. Both variants are found in public EST libraries in equal proportion and at non negligible expression levels. Moreover, we found the variant with duplication also in five private (healthy and tumoral) libraries, but neither in ERR030856, nor in a K562, while the variant without duplication is present in three private libraries and in K562. These evidences raise the possibility that this LONP1 unannotated junction may not just be due to transcriptomic noise, may be regulated, and thus functional. It is striking that such a type of read (class 5) is found in high proportion among the chimeric RNA candidates, suggesting that this LONP1 variant is not an isolated case. Larger investigations over more libraries are needed to confirm or infirm our assumptions.

References

[1] Edgren, H., Murumagi, A., Kangaspeska, S., Nicorici, D., Hongisto, V., Kleivi, K., Rye, I. H., Nyberg, S., Wolf, M., Borresen-Dale, A., and Kallioniemi, O. Identification of fusion genes in breast cancer by paired-end rna-sequencing. *Genome Biol.* **12**(1), R6 (2011). 1, 9, 10

[2] Kim, D. and Salzberg, S. L. TopHat-Fusion: an algorithm for discovery of novel fusion transcripts. *Genome Biol.* **12**(8), R72 (2011). 9, 10

[3] Flicek, P., Aken, B. L., Beal, K., Ballester, B., Caccamo, M., Chen, Y., Clarke, L., Coates, G., Cunningham, F., Cutts, T., Down, T., Dyer, S. C., Eyre, T., Fitzgerald, S., Fernandez-Banet, J., Graf, S., Haider, S., Hammond, M., Holland, R., Howe, K. L., Howe, K., Johnson, N., Jenkinson, A., Kahari, A., Keefe, D., Kokocinski, F., Kulesha, E., Lawson, D., Longden, I., Megy, K., Meidl, P., Overduin, B., Parker, A., Pritchard, B., Prlic, A., Rice, S., Rios, D., Schuster, M., Sealy, I., Slater, G., Smedley, D., Spudich, G., Trevanion, S., Vilella, A. J., Vogel, J., White, S., Wood, M., Birney, E., Cox, T., Curwen, V., Durbin, R., Fernandez-Suarez, X. M., Herrero, J., Hubbard, T. J. P., Kasprzyk, A., Proctor, G., Smith, J., Ureta-Vidal, A., and Searle, S. Ensembl 2008. *Nucleic Acids Res.* **36**(S1), D707–714 (2008). 11

Recurrent candidate chimeric RNA in LONP1 gene

GENOME chr 19 brin -1: 5' towards 3'

Positions: 5,691,988 – 5,692,012 in blue, and 5,691,992 – 5,692,016 in yellow

Read: TCAGGCCCTGTCTGGGCCAGA*ACTG**GATG***TCAGGCCCTGTCTGGGCCAGA**

repeat sequence inside the read shown in bold

Genomic locations identified by CRAC: black arrows

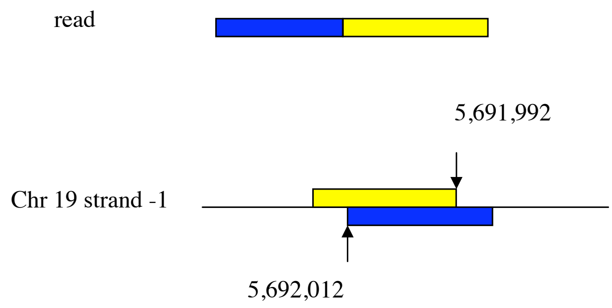


```
5692017 gatgtcaggccctgtctgggccaga           5691992
5692012      tcaggccctgtctgggccagaactg     5691992
```

BLAT alignment in two pieces

5'end

TCAGGCCCTGTCTGGGCCAGAACTGGATGTCAGGCCCTGTCTGGGCCAGA

```
1     25     25 100.0%     19     -     5691988     5692012      25

acactaccgg gagatcttcg acatcgcctt cccggacgag caggcagagg  5692063
cgctggccgt ggaacggtga cggccacccc gggactgcag gcggcggatg  5692013
TCAGGCCCTG TCTGGGCCAG AACTGagcgc tgtggggagc gcgcccggac  5691963
ctggcagtgg agccaccgag cgagcagctc ggtccagtga cccagatccc  5691913
agggacctca gtcggcttaa tcaga
```

3'end

TCAGGCCCTGTCTGGGCCAGA*ACTG**GATG***TCAGGCCCTGTCTGGGCCAGA**

```
1     25     25 100.0%     19     -     5691992     5692016      25

tggaacacta ccgggagatc ttcgacatcg ccttcccgga cgagcaggca  5692067
gaggcgctgg ccgtggaacg gtgacggcca ccccgggact gcaggcggcg  5692017
GATGTCAGGC CCTGTCTGGG CCAGAactga gcgctgtggg gagcgcgccc  5691967
ggacctggca gtggagccac cgagcgagca gctcggtcca gtgacccaga  5691917
tcccagggac ctcagtcggc ttaat
```

Figure 5: A candidate chimeric RNA involving LONP1 exons.
